# Safety and effectiveness of the early-onset sepsis calculator to reduce antibiotic exposure in at-risk newborns: a cluster-randomised controlled trial

**DOI:** 10.1016/j.eclinm.2025.103419

**Published:** 2025-08-12

**Authors:** Bo M. van der Weijden, Sanne W.C.M. Janssen, Marijke C. van der Weide, Renske J.P.M. Cornelisse-van Vugt, Gavin W. ten Tusscher, Claire A.M. Lutterman, Arvid W.A. Kamps, Carmen M. Lorente Flores, Jeroen Hol, Henriëtte van Laerhoven, Maarten Rijpert, Nadia A. Oeij, Irene A.M. Schiering, Sylvia A. Obermann-Borst, Douwe H. Visser, Lisanne M. van Leeuwen, René F. Kornelisse, Annemarie M.C. van Rossum, Merijn W. Bijlsma, Frans B. Plötz, Niek B. Achten

**Affiliations:** a Department of Paediatrics, Tergooi MC, Hilversum, the Netherlands; b Department of Paediatrics, Amsterdam UMC, Emma Children’s Hospital, Amsterdam, the Netherlands; c Department of Paediatrics, Erasmus MC, Sophia Children’s Hospital, Rotterdam, the Netherlands; d Department of Obstetrics and Gynaecology, Amsterdam UMC, University of Amsterdam, Amsterdam, the Netherlands; e Department of Paediatrics, Canisius Wilhelmina Hospital, Nijmegen, the Netherlands; f Department of Paediatrics, Dijklander Hospital, Hoorn, the Netherlands; g De Kinderpoli NH, Hoorn, the Netherlands; h Department of Paediatrics, FlevoHospital, Almere, the Netherlands; i Department of Paediatrics, Martini Hospital, Groningen, the Netherlands; j Department of Paediatrics, Máxima MC, Veldhoven, the Netherlands; k Department of Paediatrics, Northwest Clinics, Alkmaar, the Netherlands; l Department of Paediatrics, OLVG, Amsterdam, the Netherlands; m Department of Paediatrics, Zaans MC, Zaandam, the Netherlands; n Department of Paediatrics, Amstelland Hospital, Amstelveen, the Netherlands; o Department of Paediatrics, Spaarne Gasthuis, Haarlem, the Netherlands; p Care4Neo, Neonatal Patient and Parent Advocacy Organisation, Rotterdam, the Netherlands; q Department of Neonatology, Amsterdam UMC, Emma Children’s Hospital, Amsterdam, the Netherlands; r Department of Neonatal and Paediatric Intensive Care, Erasmus MC, Sophia Children’s Hospital, Rotterdam, the Netherlands

**Keywords:** Early-onset sepsis calculator, Early-onset sepsis, EOS, Neonatal sepsis calculator

## Abstract

**Background:**

Newborns are at risk for early-onset sepsis (EOS), occurring 0.2–2.0 per 1000 live births, and for antibiotic overtreatment: approximately 5–15% receive antibiotics for suspected EOS under conventional guidelines with categorical risk factor assessment. Use of the multivariate neonatal EOS calculator prediction tool can reduce overtreatment, but no trials have been conducted to compare its safety to these categorical guidelines.

**Methods:**

Between April 12th, 2022, and March 19th, 2024, we conducted an open-label, two-armed, cluster-randomised controlled trial among newborns born at ≥34 weeks’ gestational age with ≥1 EOS risk factor, comparing 10 hospitals randomised 1:1 to EOS calculator use versus categorical guideline use (ClinicalTrials.gov number: NCT05274776). The EOS calculator was slightly adapted for Dutch use. The co-primary non-inferiority outcome assessed safety using four predefined harm criteria (respiratory support, circulatory support, referral to intensive care unit, and culture-confirmed EOS). Non-inferiority was established if the upper limit of the 95% confidence interval (CI) for the relative risk did not exceed 1.5. The co-primary superiority outcome assessed the reduction of participants starting antibiotic therapy for suspected EOS within 24 h postpartum. Secondary endpoints were the duration of antibiotic therapy and the initiation of antibiotic therapy between 24 and 72 h after birth. Intention-to-treat and per-protocol analyses were performed.

**Findings:**

1830 newborns (183 per cluster) were included. At least one harm criterion was present in 64 (7.0%) of 915 in the EOS calculator arm and 134 (14.6%) of 915 in the categorical guideline arm (relative risk 0.48; 95% Cl 0.36–0.63). Antibiotics for suspected EOS were started in 66 (7.2%) of 915 in the EOS calculator arm, compared with 243 (26.6%) of 915 in the categorical guideline arm (absolute risk reduction: 19.0%, 95% CI 11.3–26.7). Median duration of antibiotics was longer in the EOS calculator arm (5.5 days, IQR 1.8–6.6) than in the categorical guideline arm (2.1 days, IQR 1.6–6.3) (P 0.0019). We found no difference in the proportion of newborns started on antibiotic therapy for suspected EOS between 24 and 72 h after birth. Adverse event rates were similar between arms. Readmission for suspected early-onset sepsis occurred three times in the EOS calculator and two times in the categorical guideline arm. Any cultures obtained at readmission remained negative, and any symptoms resolved completely.

**Interpretation:**

These trial data support safety and effectiveness of the EOS calculator for harm criteria and for the proportion of participants that started antibiotic therapy.

**Funding:**

This study was supported by SPIN, the General Paediatrics Research Network of the Dutch Association for Paediatrics, supported by het Cultuurfonds.

**Research in context**

**Evidence before this study**

Literature about the neonatal early-onset sepsis (EOS) calculator started with the publication of its fundamental models in 2011. We conducted a search in MEDLINE spanning January 1, 2011 through June 30, 2021 (when the design of this study started). We used ‘early-onset sepsis’, ‘neonatal early-onset infection’, ‘neonatal sepsis’ combined with ‘sepsis risk model’, ‘predictive model’, ‘neonatal early-onset sepsis calculator’, ‘neonatal sepsis calculator’, ‘early-onset sepsis calculator’, and ‘sepsis risk calculator’, revealing 65 relevant publications. Of these publications, 42 studies evaluated the EOS calculator, with 11 of these examining actual implementation of the EOS calculator. All studies were observational, some with a control group in a before-and-after design, and most were limited to newborns of at least 35 weeks’ gestational age. A systematic review and meta-analysis of observational studies indicated a reduction in antibiotic use of up to 44%, without evidence of adverse effects, albeit with limited power for the latter. There are no known implementation studies in which the use of the EOS calculator was ineffective or unsafe. Studies retrospectively applying it to larger datasets have suggested the possibility of delay in diagnosis or treatment but exhibit a high risk of bias.

**Added value of this study**

In this randomised controlled trial comparing an adapted EOS calculator to the conventional categorical approach for the management of EOS risk, we determined the safety outcome reflecting the severity of illness in newborns with suspected EOS. The EOS calculator was non-inferior to the Dutch categorical guideline regarding safety, and reduced antibiotic exposure.

**Implications of all the available evidence**

This study added to existing observational evidence, that the EOS calculator may be a safe and effective way to reduce antibiotic overtreatment in newborns. However, research also shows that all available EOS risk management strategies, including the EOS calculator, initially classify a considerable portion of EOS cases as low risk. Implementation should therefore occur carefully and only in conjunction with appropriate vigilance for early signs of EOS.

## Introduction

Early-onset sepsis (EOS) is a serious bacterial infection, causing morbidity and mortality in newborns.^1^ It is proven using cultures in approximately 0.2–2.0 per 1000 live births, but suspected considerably more often.^2^, ^3^, ^4^ The challenge of timely recognition of EOS and the risk of serious consequences from untreated EOS have resulted in a low threshold for initiating empiric antibiotic treatment, reflected in guidelines for EOS risk management. Guidelines such as those of the National Institute for Health and Care Excellence (NICE), contain categorical strategies based on maternal risk factors and neonatal clinical signs of infection.^5^ However, implementation of these guidelines has led to increased antibiotic use.^6^ Approximately 5–15% of newborns receive empiric intravenous antibiotic treatment as per NICE recommendation,^7^ a rate up to 250 times higher than the incidence of proven EOS. This overtreatment disrupts early family life, involves painful procedures, and invokes unnecessary hospitalisations and associated costs. Long-term risks include negative impacts on the microbiome and immune system, linked to chronic disorders such as asthma and allergy.^8^, ^9^, ^10^, ^11^ On a societal level, antibiotic overtreatment contributes to antimicrobial resistance and healthcare costs.^12^

In 2011, Kaiser Permanente introduced the EOS calculator to reduce antibiotic overtreatment in newborns of ≥34 weeks’ gestational age.^13^, ^14^, ^15^ Using a multivariate approach with continuous variables instead of categorical assessment of risk factors, this prediction tool calculates a newborn’s individual EOS risk based on both maternal and neonatal factors. It subsequently stratifies newborns into risk groups with specific treatment recommendations. Observational studies show that implementation of the EOS calculator helps reduce antibiotic exposure by approximately 44% compared with conventional guidelines.^16^^,^^17^

Confidence in the safety of the EOS calculator is pivotal for its implementation.^18^ Although there are no implementation studies demonstrating the EOS calculator to be unsafe, concerns have been raised about a potentially lower sensitivity to detect proven EOS cases compared with categorical guidelines.^18^, ^19^, ^20^ The EOS calculator’s ability to identify EOS depends largely on clinical observation of developing symptoms,^21^ and there is a paucity of data on whether this aspect leads to delayed treatment, with or without clinical significance. Furthermore, the EOS calculator was validated using culture-confirmed sepsis. It may be less sensitive for potential cases of culture-negative sepsis,^4^^,^^22^ meaning implementation could possibly delay treatment of such cases. Illustrating these uncertainties, the latest NICE guidelines require prospective monitoring during EOS calculator usage.^23^

To adequately balance the burden of overtreatment with risk of EOS, data from randomised studies are needed to inform clinicians on the actual effectiveness and safety of the EOS calculator approach. Therefore, we initiated this controlled trial to investigate whether the EOS calculator can safely reduce antibiotic exposure in newborns with suspected EOS, compared with the Dutch adaptation of the NICE categorical guideline.

## Methods

### Study design

This was an open-label, two-armed, parallel cluster-randomised controlled trial, with a co-primary non-inferior and superior outcome. We used a cluster-randomised rather than individually randomised design because of the urgency associated with the decision to start antibiotics, and to prevent contamination bias that could occur with simultaneous open-label use of multiple protocols in a single hospital. Data were collected from 10 non-tertiary hospitals in the Netherlands from April 12th, 2022, to March 19th, 2024. Each participating hospital served as an individual cluster. This trial was prospectively registered at ClinicalTrials.gov (NCT05274776). The trial protocol has been published.^24^ The CONSORT reporting guidelines for cluster-randomised trials were used for reporting.^25^

### Ethics

This trial was approved by the medical ethics review committee of the Amsterdam University Medical Center (NL78203.018.21; January 14th, 2022). Informed consent was obtained for all participants.

### Clusters and participants

Cluster eligibility assessment and selection were performed prior to randomisation and restricted to non-tertiary hospitals with a maternity ward. This restriction prevented possible bias that would occur if the high-risk populations from a tertiary hospital would be randomised to one of the arms. It also ensured feasibility to achieve the sample size within a cluster, since the target population is predominantly admitted in non-tertiary hospitals. Eligible hospitals were approached for participation by the principal investigator and further selected based on existing research infrastructure to support feasibility. Newborns were eligible for participation if the gestational age was ≥34 weeks and at least one EOS risk factor was present within 24 h after birth (EOS calculator use is valid for 24 h postpartum). If EOS risk factors occurred after the first 24 h of life, the categorical guideline was applied regardless of cluster randomisation. Eligibility criteria were the same for both arms, with EOS risk factors (both maternal and neonatal) for inclusion being congruent with the categorical guideline (Appendix pp 2–3).^26^ We considered these factors absent if their presence was not documented during clinical practice. Exclusion criteria were the presence of major congenital anomalies or a significant language barrier.^24^ Within 24 h of birth, local research personnel approached the parents of eligible newborns. The parents received oral and written explanations and provided written informed consent for the collection and use of data. Based on equipoise, both the categorical guideline and the EOS calculator were considered standard care. Therefore, consistent with Dutch regulations, consent was required for data collection only.

### Randomisation and masking

Participating hospitals were randomised to either the categorical guideline or EOS calculator for suspected EOS management, which was subsequently adopted as standard of care. The Dutch categorical guideline is an adaptation of the 2012 NICE guideline.^23^^,^^26^ It uses eight maternal and 15 neonatal risk factors, categorised as non-red or red flags (Appendix, pp 2–3). Antibiotic treatment is recommended if there is at least one red flag or two non-red flags. Observation is recommended if there is one non-red flag (Appendix, p 4). The EOS calculator combines maternal risk factors and physical examination findings to quantitatively estimate EOS risk, and to guide clinical decision making on whether to initiate empiric antibiotics, initiate enhanced observation using vital signs every 3 h for 24 h, or opt for routine monitoring (Appendix, pp 2–3, 5).^15^^,^^22^ Cluster randomisation for either the categorical guideline or EOS calculator occurred at the hospital level using a 1:1 randomisation scheme making use of a computer-generated algorithm, resulting in five hospitals continuing categorical guideline use and five implementing the EOS calculator. Randomisation was executed by an independent methodologist in PASS 2020. Hospitals and physicians were not blinded due to the nature of this trial.

### Procedures

In the hospitals, all newborns were screened for EOS risk. For those with at least one risk factor, clinical evaluations were conducted within 4 h after birth, and recommendations of either the categorical guideline or the EOS calculator regarding observation or the initiation of antibiotic therapy were followed. This included a clinical observation period of at least 12 h for the categorical guideline arm, and a minimum of 24 h for the EOS calculator arm, in accordance with the recommendations of each strategy, respectively. In both arms, if antibiotics were started, local regime was used, and a blood culture was obtained before the start of antibiotics. A reduction in empiric antibiotic treatment might increase the risk of treated newborns truly being infected. Therefore, while discontinuation of antibiotic treatment for the categorical guideline arm followed the categorical guideline, this decision was left to the discretion of the treating physician in the EOS calculator arm. Observation for both arms included monitoring vital parameters (heart rate, respiratory rate, and temperature) every 3 h by nursing staff, along with a minimum of two physical examinations by a physician (conducted within 4 h after birth and prior to discharge). Before discharge, parents were instructed to contact the hospital if there were signs of infection within the first 14 days of life.

Hospitals randomised to the EOS calculator were supplied with a smartphone application incorporating the EOS calculator prediction tool, slightly adapted for Dutch clinical practice in two ways. The original EOS calculator tool recommendations include a blood culture without the start of empiric antibiotics, a practice of unclear clinical value,^22^ which was therefore replaced with clinical observation. In addition, amoxicillin/clavulanic acid was specifically specified as a broad-spectrum intrapartum antibiotic prophylaxis, as it is commonly used in the Netherlands and considered locally effective prophylaxis against dominant EOS pathogens.^27^ Data were collected by research nurses at participating hospitals and stored in a digital database (Castor EDC, Ciwit B.V.). Independent monitors conducted source data verification and assessed trial procedures at each site at least once a year, sampling 10% of the data.

### Outcomes

The co-primary non-inferiority outcome was measured as the occurrence of one or more of four predefined harm criteria: the need for any form of respiratory support (invasive ventilation, continuous positive airway pressure, high-flow nasal cannula, low-flow oxygen) after the first 30 min but during the first week of life, the administration of an intravascular fluid bolus for haemodynamic instability due to sepsis, referral to a neonatal intensive care unit for sepsis treatment, and the incidence of proven EOS. Proven EOS was defined as a pathogenic bacterial species isolated from a blood or cerebrospinal fluid culture obtained within 72 h after birth. Cultures were processed and analysed according to the local protocols of the participating hospitals, including interpretation of possible contamination as determined by the local clinical team; if only contaminants were isolated, cultures were considered negative for this study. To assess the co-primary superiority outcome, we measured the proportion of participants in whom antibiotic therapy for suspected or proven EOS within the first 24 h after birth was initiated. Two secondary endpoints were defined: the total duration of antibiotic therapy, and the proportion of antibiotic therapy initiated for suspected or proven EOS when symptoms arose between 24 and 72 h after birth.

All adverse events were recorded up to day 14 after birth to capture complications related to initial EOS management, including changes in EOS course or readmission. If a newborn required continued antibiotic therapy beyond day 14 due to EOS or a serious adverse event, documentation of adverse events continued until treatment was concluded. An independent data safety monitoring board monitored the trial.

### Statistical analysis

Sample size calculations and margins for the study considered both non-inferiority and superiority outcomes, considered preceding research, and were published previously in the protocol.^24^ In brief, given an expected power of 80%, an intracluster correlation coefficient of 0.0025 (estimated without specific source given absence of relatable cluster-randomised controlled trials), and an alpha level of 0.05 across 10 hospitals, use of the EOS calculator was considered non-inferior if the upper bounds of the 95% CI of the relative risk of harm criteria presence did not exceed 1.5 (equalling an absolute non-inferiority margin of 5% with an expected 10% presence of harm criteria in the control arm), requiring a sample size of 1640 (164 per cluster). For superiority, detecting a reduction from 40% anticipated participants receiving antibiotic therapy under the categorical guidelines to 25% using the EOS calculator (absolute risk reduction of 15%),^28^ required a sample size of 330. The total sample size was determined by the larger estimate and adjusted for a 10% dropout rate, resulting in 1830 newborns (2 × 915) to be included, equating 183 per cluster.

Both arms included five clusters for all analyses. Analyses were performed according to a prespecified statistical analysis plan, with documentation of any protocol violations. The primary analyses were conducted as intention-to-treat, with per-protocol as secondary analyses. A mixed model fitted with the Gauss-Hermite quadrature method and cluster as random effect was used. Relative risk was calculated utilising a binomial distribution with a log link, risk difference with an identity link. The per-protocol analyses excluded participants in case of crossover to the other arm, non-adherence to the assigned guideline, or the use of data that was inconsistent with the electronic health record. Because of the small number of clusters, in a sensitivity analysis a cluster summary analysis using summary statistics for each cluster was conducted to check the robustness of the primary analysis. To demonstrate effectiveness of the EOS calculator, both non-inferiority for harm criteria and superiority for the initiation of antibiotic therapy were required.^24^ Regarding non-inferiority, relative risk was used as effect size. For superiority, both relative risk and risk difference were used. For both co-primary outcomes, we calculated 95% confidence intervals (CIs), P-values, and intraclass correlation coefficients (ICC; using the intention-to-treat population). Interim safety analyses were performed by the data safety monitoring board after data collection for the first 200 participants in each arm and again at 450 participants per arm. No interim analyses on efficacy were performed.

Dichotomous secondary outcomes were analysed using a generalised estimating equations (GEE) model with a log link, binomial distribution, and including centre as a cluster variable, while continuous secondary outcomes were assessed with either a linear GEE model to estimate mean differences, when normally distributed, or with a k samples median test when skewed. Analyses were performed using IBM SPSS Statistics (version 28), Stata/SE (version 17.0), and SAS (version 9.4). Any missing data for the primary analyses were completed by additional review of the electronic health care record. In case of missing data for secondary analyses, these participants were omitted from these analyses, with missing data listed in each table accordingly.

### Role of the funding source

This study was supported by SPIN, the General Paediatrics Research Network of the Dutch Association for Paediatrics, supported by het Cultuurfonds. SPIN had no role in study design, data collection, data analysis, data interpretation, or writing of the report.

## Results

### Participants

**Fig. 1:**
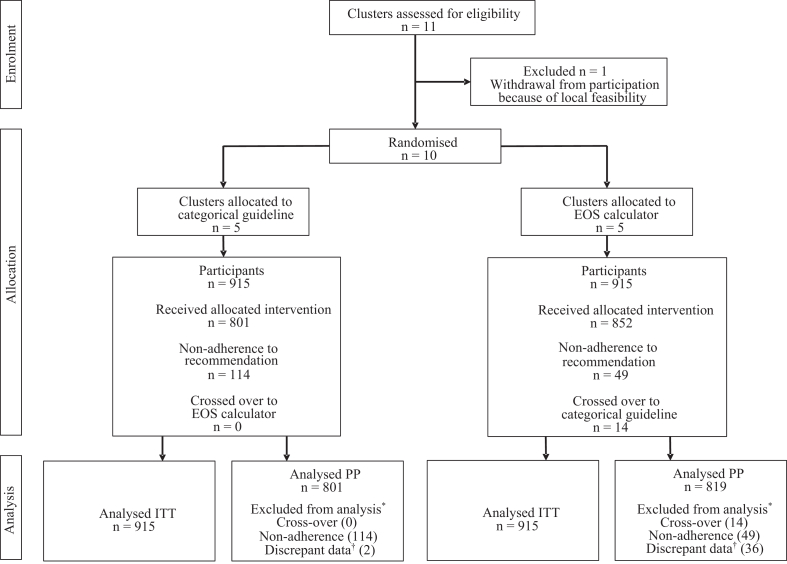
CONSORT Flow Chart of Participants. EOS = early-onset sepsis. ITT = intention-to-treat. PP = per-protocol. ∗Not mutually exclusive. ^†^Data important for EOS risk management but inconsistent with the electronic health record.

Among approximately 27,500 births of ≥34 weeks gestational age, an estimated 2500 newborns with one or more EOS risk factors were approached for consent (data extrapolated from available registers and screening logs). During a median inclusion time of 15 months (interquartile range (IQR) 13–20), each participating hospital included 183 newborns, resulting in a total of 1830 newborns, 915 in each arm (Fig. 1). All newborns were included in the intention to treat analysis. In total 210 (11.5%) of 1830 were excluded from the per-protocol analysis (Appendix, p 7); 96 (10.5%) of 915 were excluded from the EOS calculator arm and 114 (12.5%) of 915 from the categorical guideline arm. The most common reason for exclusion from the per-protocol analysis was non-adherence to the treatment advice of the guideline (49 (51.0%) of 96 in the EOS calculator arm, and 114 (100.0%) of 114 in the categorical guideline arm). The reason for non-adherence was mostly unknown (130 (79.8%) of 163), followed by ‘good clinical condition despite the risk factors’ (20 (12.3%) of 163). Antibiotics were given when treatment was not advised in 40 (81.6%) of 49 in the EOS calculator arm and 52 (45.6%) of 114 in the categorical guideline arm, respectively (Appendix, p 7). Antibiotics were not given when treatment was advised in 9 (18.4%) of 49 in the EOS calculator arm and 62 (54.4%) of 114 in the categorical guideline arm, respectively (Appendix, p 7).

### Patient characteristics

General baseline characteristics were well-balanced between the arms, including median gestational age (Table 1). The median Apgar score at 5 min post-partum was 10. Eighty percent of newborns were born via vaginal delivery.

**Table 1:** Patients characteristics.

Baseline characteristics	Categorical guideline N = 915	EOS calculator N = 915
**Child**		
Male sex, n (%)	503 (55.0)	464 (50.7)
Gestational age weeks, mean (SD)	38.51 (2.06)	38.60 (2.02)
34–36.9 weeks, n (%)	191 (20.9)	181 (19.8)
37–42 weeks, n (%)	724 (79.1)	734 (80.2)
Apgar score 1 min, median (IQR)	9 (8–9)^a^	8 (8–9)^b^
Apgar score 5 min, median (IQR)	10 (9–10)^c^	10 (9–10)^d^
**Mother**		
Mode of delivery		
Caesarean section, n (%)	192 (21.0)	174 (19.0)
Vaginal delivery, n (%)	723 (79.0)	741 (81.0)
Maternal risk factors		
Parenteral antibiotic treatment, n (%)	37 (4.2)^e^	40 (4.4)^f^
Suspected or confirmed infection in sibling from multiple pregnancy, n (%)	1 (0.1)	2 (0.2)
Invasive GBS in previous neonate born to mother, n (%)	19 (2.1)	22 (2.4)
GBS status evaluation, n (%)	913 (99.8)^d^	902 (98.6)^g^
Positive, n (%)	81 (8.9)	133 (14.7)
Negative, n (%)	173 (18.9)	120 (13.3)
Unknown before first evaluation, n (%)	659 (72.2)	649 (72.0)
Spontaneous preterm birth, n (%)	165 (18.0)	170 (18.6)
Duration rupture of membranes (hours), median (IQR)	21.0 (8.0–38.0)^h^	21.0 (9.0–45.0)^i^
Prelabour rupture of membranes ≥24 h in a term birth, n (%)	360 (50.6)^j^	373 (51.2)^a^
Prelabour rupture of membranes ≥18 h in a preterm birth, n (%)	66 (34.9)^d^	60 (33.3)^c^
Highest maternal antepartum temperature, median (IQR)	37.5 (37.0–38.1)^k^	37.4 (36.9–38.1)^g^
Intrapartum fever >38 °C or (suspected) chorioamnionitis, n (%)	287 (31.4)^d^	300 (32.9)^b^
Type of intrapartum antibiotics		
Broad spectrum ≥4 h prior to birth, n (%)	56 (6.1)^d^	78 (8.5)^c^
Broad spectrum 2–4 h prior to birth, n (%)	67 (7.3)^d^	97 (10.6)^c^
GBS specific ≥2 h prior to birth, n (%)	162 (17.7)^d^	147 (16.1)^c^
No antibiotics prior to birth, n (%)	628 (68.8)^d^	592 (64.8)^c^
Neonatal risk factors at inclusion		
Respiratory distress starting >4 h after birth, n (%)	14 (1.5)	2 (0.2)^c^
Neonatal epileptic seizures, n (%)	0 (0.0)	1 (0.1)^d^
Need for mechanical ventilation in a term neonate, n (%)	3 (0.4)	1 (0.1)
Signs of shock, n (%)	0 (0.0)	0 (0.0)^d^
Altered behaviour with regard to responsiveness or muscle tone, n (%)	38 (4.2)	21 (2.3)
Feeding difficulties, n (%)	45 (4.9)	73 (8.0)^l^
Apnoea and bradycardia, n (%)	6 (0.7)	1 (0.1)^c^
Signs of respiratory distress, n (%)	253 (27.7)	118 (12.9)^c^
Hypoxia, n (%)	93 (10.2)	55 (6.0)
Neonatal encephalopathy, n (%)	0 (0.0)	0 (0.0)
Need for cardiopulmonary resuscitation, n (%)	1 (0.1)	0 (0.0)
Need for mechanical ventilation in a preterm neonate, n (%)	4 (2.1)	3 (1.7)
Persistent pulmonary hypertension, n (%)	4 (0.4)	3 (0.3)
Unexplained temperature abnormality, n (%)	102 (11.3)^m^	72 (8.1)^e^
Local signs of infection, n (%)	13 (1.4)	3 (0.3)^l^

EOS = early-onset sepsis. GBS = Group B Streptococcus. IQR = interquartile range. SD = standard deviation.

a 5 missing.

b 4 missing.

c 1 missing.

d 2 missing.

e 27 missing.

f 7 missing.

g 13 missing.

h 14 missing.

i 6 missing.

j 12 missing.

k 87 missing.

l 3 missing.

m 15 missing.

### Maternal and neonatal risk factors

Maternal risk factors were well balanced among the arms. The most frequently encountered maternal risk factor, in both arms, was prolonged prelabour rupture of membranes (50.6% and 51.2% for the categorical guideline and the EOS calculator respectively, Table 1). Recorded maternal GBS colonisation status was unknown (not tested or no result at baseline) for most newborns (72.1%). A positive maternal GBS status was recorded in 11.8%, and a negative status in 16.1%, respectively. The most common neonatal risk factor, ‘signs of respiratory distress’, occurred less often (118 of 915, 12.9%) in the EOS calculator arm than in the categorical guideline arm (253 of 915, 27.7%).

### Safety

The intention-to-treat analysis contained data from 1830 newborns for the co-primary non-inferiority outcome. Fewer harm criteria occurred among newborns in the EOS calculator arm compared with the categorical guideline arm (Table 2). At least one harm criterion was present in 64 (7.0%) of 915 newborns in the EOS calculator arm, compared with 134 (14.6%) of 915 newborns in the categorical guideline arm (relative risk 0.48; 95% CI 0.36–0.63), indicating non-inferiority and an accompanying protective effect (ICC 0.015). The cluster summary analysis showed similar results (relative risk 0.48; 95% CI 0.38–0.61). In both arms, the need for respiratory support was the most frequent criterion (62 (6.8%) of 915 in the EOS calculator arm, 133 (14.5%) of 915 in the categorical guideline arm). Respiratory support was most often supplied as continuous positive airway pressure. Tertiary referral for suspected EOS occurred in zero newborns in the EOS calculator arm, and in five (0.5%) of 915 in the categorical guideline arm. Proven EOS occurred in two (0.2%) of 915 and three (0.3%) of 915 newborns in these arms, respectively. All five received antibiotic treatment directly after birth. Three EOS cases were caused by Group B *Streptococcus*, and two by *Escherichia coli*. Per-protocol analysis for the primary outcomes revealed comparable results (Table 2). Given the baseline difference between study arms, a post-hoc sensitivity analysis was performed correcting for respiratory distress at baseline. This showed that the condition for non-inferiority was still met (relative risk for presence of at least one harm criterion 0.74; 95% CI 0.60–0.92, P 0.0059).

**Table 2:** Co-primary outcomes and related secondary outcomes.

Non-inferiority & Superiority	Categorical guideline N = 915	EOS calculator N = 915	RR (95% CI)	P-value
**Co-primary non-inferiority outcome**				
Presence of at least one harm criterium, n (%) ITT	134 (14.6)	64 (7.0)	0.48 (0.36–0.63)	0.0000
Presence of at least one harm criterium, n (%) PP	100 (12.5)	42 (5.1)	0.41 (0.29–0.58)	0.0000
Harm criteria				
Need for respiratory support^d^, n (%)	133 (14.5)	62 (6.8)	0.47 (0.39–0.56)	0.0000
Invasive/Mechanical ventilation, n (%)	7 (0.8)	4 (0.4)		
CPAP, n (%)	117 (12.8)	63 (6.9)		
High flow (>2 L/min.), n (%)	31 (3.4)	9 (1.0)		
Low flow (≤2 L/min.), n (%)	33 (3.6)	15 (1.6)		
Need for supplemental oxygen^d^, n (%)	93 (10.2)	55 (6.0)	0.59 (0.41–0.86)	0.0059
Intravascular fluid bolus for haemodynamic instability (related to EOS), n (%)	0 (0)	0 (0)		
NICU referral for sepsis treatment, n (%)	5 (0.5)	0 (0)		
Proven EOS, n (%)	3 (0.3)	2 (0.2)	0.67 (0.18–2.42)	0.5379
Blood culture positive, n (%)	3 (0.3)	2 (0.2)		
Pathogen: *E. coli*, n (%)	2 (0.2)	0 (0)		
Pathogen: GBS, n (%)	1 (0.1)	2 (0.2)		
Pathogen: Other, n (%)	0 (0)	0 (0)		
Cerebrospinal fluid culture positive, n (%)	1 (0.1)	0 (0)		
Pathogen: *E. coli*, n (%)	1 (0.1)	0 (0)		
**Co-primary superiority outcome**				
(suspected) EOS treatment started within 24 h, n (%) ITT	243 (26.6)	66 (7.2)	RR: 0.28 (0.18–0.43) RD: −19.01 (−26.72 to 11.30)	0.0000
(suspected) EOS treatment started within 24 h, n (%) PP	195 (24.3)	31 (3.8)	RR: 0.16 (0.09–0.27) RD: −20.27 (−27.14 to 13.40)	0.0000
**Secondary outcomes**				
Antibiotic treatment				
Antibiotic treatment (total), n (%)	259 (28.3)	73 (8.0)	0.28 (0.21–0.38)	0.0000
Started directly after first evaluation, n (%)	210 (23.0)	42 (4.6)		
0–12 h after birth, n (%)	215 (23.5)	56 (6.1)		
12–24 h after birth, n (%)	28 (3.1)	10 (1.1)		
If symptoms started 24–72 h after birth, n (%)	16 (1.7)	7 (0.8)		0.1291
Total duration of treatment in days, median (IQR)	2.1 (1.6–6.3)	5.5 (1.8–6.6)		0.0019
Sum of duration of treatment in days, median (IQR)	161.8 (155.3–234.6)	58.4 (53.6–70.7)		0.0000^c^

CPAP = continuous positive airway pressure. *E. coli* = *Escherichia coli*. EOS = early-onset sepsis. GBS = Group B Streptococcus. IQR = interquartile range. ITT = intention-to-treat. NICU = neonatal intensive care unit. PP = per-protocol. RR = relative risk. RD = risk difference. ^a^1 missing. ^b^2 missing.

c Analysed on cluster level, using the k-samples median test.

d Durations included in the Appendix (Appendix p 9).

### Use of antibiotics

The intention-to-treat analysis contained data from all 1830 newborns for the superiority outcome. Initiation of antibiotic therapy for suspected EOS within 24 h after birth was lower in the EOS calculator arm (66 (7.2%) of 915), compared with the categorical guideline arm (243 (26.6%) of 915). This absolute risk reduction of 19.0% (corrected for intracluster correlation; 95% CI 11.3–26.7; cluster summary analysis: risk difference 19.3, 95% CI 9.6–29.1) demonstrated superiority in favour of the EOS calculator arm (Table 2) with an ICC of 0.096. Between 24 and 72 h after birth, there was no difference in the proportion of newborns started on antibiotic therapy for suspected EOS. Median duration of antibiotics was longer in the EOS calculator arm (5.5 days, IQR 1.8–6.6) than in the categorical guideline arm (2.1 days, IQR 1.6–6.3) (P 0.0019). Post-hoc analysis showed that the median sum of antibiotic treatment days among clusters was lower in the EOS calculator arm (58.4 days) compared with the categorical guideline arm (161.8 days) (P < 0.001, k samples median test). Per-protocol analysis revealed comparable results (Table 2).

### Clinical evaluations

On average, in both arms the first clinical evaluation occurred 1.7 h after birth (Table 3). During the observation period, a minority of newborns (225 (24.6%) of 916 in the EOS calculator arm and 108 (11.8%) of 915 in the categorical guideline arm) developed potential signs of infection during ongoing clinical evaluation (Table 4). During ongoing clinical evaluation, respiratory symptoms where less often present in the EOS calculator arm compared with the categorical guideline arm. Infection biomarkers were determined in 72 (7.9%) of 915 newborns in the EOS calculator arm, compared with 257 (28.1%) of 915 in the categorical guideline arm (Table 5, P < 0.001). Results showed that a first C-reactive protein value was obtained later (19.8 h versus 7.1 h postpartum, P < 0.001), and slightly more elevated in the EOS calculator arm (7.9 mg/L (range, 2.3–27.8) versus 2.1 mg/L (range, 1.0–10.0), P 0.0015).

**Table 3:** Neonatal clinical condition at first evaluation.

Neonatal clinical condition	Categorical guideline N = 915	EOS calculator N = 915	RR (95% CI)	P-value
**First evaluation of newborn (hours after birth), median (IQR)**	1.7 (0.9–3.1)^a^	1.8 (0.9–2.9)^b^		0.0446
**Respiratory**				
Respiratory rate, mean (SD)	45.9 (13.9)^d^	42.9 (11.6)^e^	−6.07 (−10.77 to −1.34)^s^	0.0112^f^
Respiratory distress symptoms, n (%)	253 (27.7)	118 (12.9)^c^	0.47 (0.33–0.66)	0.0000^c^
Grunting sound, n (%)	102 (11.1)^g^	44 (4.8)		
Flaring nostrils, n (%)	47 (5.1)^g^	24 (2.6)		
Retractions, n (%)	91 (9.9)^g^	35 (3.8)		
Tachypnoea, n (%)	163 (19.7)^d^	79 (9.4)^e^		
Duration of tachypnoea, median (IQR)	5.5 (2.8–24.0)	4.8 (3.0–11.5)		0.2906
Duration of respiratory distress symptoms, median (IQR)	10.0 (3.9–32.1)^h^	7.1 (1.8–25.9)^i^		0.1704^j^
Causes of respiratory insufficiency^r^, n (%)	50 (5.5)	17 (1.9)	0.34 (0.17–0.71)	0.0039
Transition disorder, n (%)	33 (3.6)	8 (0.9)		
Meconium aspiration syndrome, n (%)	6 (0.7)	2 (0.2)		
Congenital hypotonia, n (%)	0 (0)	1 (0.1)		
Diaphragmatic hernia, n (%)	0 (0)	0 (0)		
Other, n (%)	25 (2.7)	9 (1.0)		
Need for respiratory support, n (%)	139 (15.2)	71 (7.8)	0.51 (0.38–0.69)	0.0001
Need for surfactant therapy, n (%)	4 (0.4)	0 (0)		
Frequency of surfactant therapy, mean (SD)	1.5 (0.6)	0 (0)		
Need for nitric oxide, n (%)	1 (0.1)	0 (0)		
Need for extracorporeal membrane oxygenation, n (%)	0 (0)	0 (0)		
**Haemodynamic**				
Heart rate, mean (SD)	138.1 (16.6)^k^	140.3 (16.4)^l^	2.17 (−0.84 to 5.18)^s^	0.1576^m^
Tachycardia, n (%)	91 (10.8)^k^	117 (13.8)^l^	1.27 (0.91–1.78)	0.1574^m^
Duration of tachycardia (hrs), median (IQR)	3.0 (1.4–5.0)^n^	3.0 (1.7–5.0)^c^		0.8982^o^
Need for fluid bolus or inotropics for suspected EOS, n (%)	0 (0)	0 (0)		
**Temperature**				
Temperature, median (IQR)	37.0 (36.7–37.4)^n^	37.0 (36.7–37.4)^p^		0.4765^q^
Duration of abnormal temperature (hrs), median (IQR)	3.4 (1.9–6.7)	3.5 (2.4–7.2)		0.1427

CI = confidence interval. EOS = early-onset sepsis. hrs = hours. IQR = interquartile range. RR = relative risk.

a 24 missing.

b 13 missing.

c 1 missing.

d 87 missing.

e 75 missing.

f 162 missing.

g 3 missing.

h 115 missing.

i 61 missing.

j 176 missing.

k 76 missing.

l 67 missing.

m 143 missing.

n 15 missing.

o 16 missing.

p 26 missing.

q 41 missing.

r Not mutually exclusive.

s Mean difference.

**Table 4:** Risk factors and neonatal clinical condition during observation.

Risk factors and neonatal clinical condition during observation (including baseline)	Categorical guideline N = 915	EOS calculator N = 915	RR (95% CI)	P-value
**Change in neonatal condition, n (%)**	108 (11.8)	225 (24.6)		0.0208
**Maternal risk factors**				
GBS status, n (%)	614 (67.3)^a^	710 (78.7)^b^	1.17 (0.98–1.39)	0.0779^c^
Positive after evaluation, n (%)	48 (5.2)	72 (7.9)		0.1874
Negative after first evaluation, n (%)	294 (32.1)	338 (36.9)		0.5564
Unknown after first evaluation, n (%)	573 (62.6)	505 (55.2)		0.4311
**Neonatal risk factors**				
Respiratory distress starting >4 h after birth, n (%)	32 (3.5)	17 (1.9)	0.53 (0.27–1.06)	0.0707
Neonatal epileptic seizures, n (%)	0 (0)	2 (0.2)		
Need for mechanical ventilation in a term neonate, n (%)	3 (0.4)	1 (0.1)	0.57 (0.06–5.60)	0.6307
Signs of shock, n (%)	0 (0)	0 (0)		
Need for cardiopulmonary resuscitation, n (%)	0 (0)	0 (0)		
Altered behaviour with regard to responsiveness or muscle tone, n (%)	46 (5.0)	45 (4.9)	0.98 (0.50–1.92)	0.4989
Feeding difficulties, n (%)	65 (7.1)	132 (14.5)^d^	2.04 (0.93–4.49)	0.0771^d^
Apnoea and bradycardia, n (%)	21 (2.7)^e^	30 (4.5)^f^	1.67 (1.10–2.30)	0.0171^g^
Signs of respiratory distress, n (%)	274 (29.9)	169 (18.5)	0.62 (0.43–0.89)	0.0095
Hypoxia, n (%)	93 (10.2)	55 (6.0)	0.59 (0.41–0.86)	0.0059
Neonatal encephalopathy, n (%)	0 (0)	0 (0)		
Need for cardiopulmonary resuscitation, n (%)	1 (0.1)	0 (0.0)		
Need for mechanical ventilation in a preterm neonate, n (%)	4 (50.3)	3 (47.8)	0.75 (0.42–1.35)	0.3399
Persistent pulmonary hypertension, n (%)	4 (0.4)	3 (0.3)	0.75 (0.17–3.24)	0.7002
Unexplained temperature abnormality, n (%)	116 (12.5)^c^	118 (12.7)^h^	1.03 (0.54–1.96)	0.9287^i^
Local signs of infection, n (%)	17 (1.9)	7 (0.8)^d^	0.41 (0.13–1.33)	0.1373^d^
Respiratory				
Respiratory rate, mean (SD)∗	50.2 (16.6)^j^	45.3 (15.1)^k^	−4.84 (−9.80 to 0.11)^u^	0.0554^l^
Respiratory distress symptoms	274 (29.9)	169 (18.5)	0.62 (0.43–0.89)	0.0095
Grunting sound, n (%)	111 (12.1)	50 (5.5)		
Flaring nostrils, n (%)	57 (6.2)	30 (3.3)		
Retractions, n (%)	104 (11.4)	46 (5.0)		
Tachypnoea, n (%)	193 (21.1)	137 (15.0)		
Duration of tachypnoea, median (IQR)	6.9 (3.0–27.5)	5.4 (3.0–18.2)^d^		0.4464^d^
Duration of respiratory distress symptoms, median (IQR)	12.0 (4.0–39.6)^m^	8.8 (3.1–26.9)^m^		0.4848^n^
Haemodynamic				
Heart rate, mean (SD)	138.1 (16.8)^o^	140.8 (18.6)^p^	2.64 (−0.60 to 5.87)^u^	0.1105^q^
Tachycardia, n (%)	93 (11.1)^o^	133 (15.7)^p^	1.42 (1.05–1.91)	0.0238^q^
Duration of tachycardia, median (IQR)	3.0 (1.4–5.0)^a^	3.2 (1.8–5.6)^r^		0.6069^d^
Need for fluid bolus or inotropics for suspected EOS, n (%)	0 (0)	0 (0)		
Temperature				
Temperature, median (IQR)	37.0 (36.6–37.4)^b^	36.9 (36.5–37.4)^s^		0.3448^t^
Duration of abnormal temperature, median (IQR)	3.4 (2.0–6.8)	3.0 (1.8–5.4)		0.1864

CI = confidence interval. EOS = early-onset sepsis. GBS = Group B Streptococcus. IQR = interquartile range. RR = relative risk. SD = standard deviation.

a 2 missing.

b 13 missing.

c 15 missing.

d 3 missing.

e 134 missing.

f 254 missing.

g 388 missing.

h 27 missing.

i 41 missing.

j 81 missing.

k 70 missing.

l 151 missing.

m 9 missing.

n 18 missing.

o 75 missing.

p 66 missing.

q 141 missing.

r 1 missing.

s 25 missing.

t 38 missing.

u Mean difference.

**Table 5:** Laboratory results.

Laboratory results	Categorical guideline N = 915	EOS calculator N = 915	P-value
Infection markers, n (%)	257 (28.1)	72 (7.9)	0.0000
Total 1st CRP, n (%)	257 (28.1)	69 (7.5)	0.0000
1st CRP level (mg/L), median (IQR)	2.1 (1.0–10.0)^a^	7.9 (2.3–27.8)^a^	0.0015^b^
Time to 1st CRP level (hrs), median (IQR)	7.1 (4.0–15.3)	19.8 (7.8–28.5)^b^	0.0000^b^
Total 2nd CRP, n (%)	223 (24.4)	39 (4.3)	0.0780
2nd CRP level (mg/L), median (IQR)	9.0 (2.0–29.0)	11.0 (3.0–38.0)	0.2244
Time to 2nd CRP level (hrs), median (IQR)	27.4 (24.0–39.0)	39.0 (30.4–48.4)	0.0000
Total 1st PCT, n (%)	70 (7.8)	1 (0.1)	0.0146
1st PCT level (ng/mL), median (IQR)	2.6 (1.2–7.7)^b^	10.7	0.9650
Time to 1st PCT level (hrs), median (IQR)	6.7 (6.3–7.7)	13.7	0.9887
Total 2nd PCT, n (%)	53 (5.8)	0 (0)	
2nd PCT level (ng/mL), median (IQR)	4.9 (3.0–13.5)		
Time to 2nd PCT level (hrs), median (IQR)	24.7 (24.0–26.3)		
Total 1st WBC, n (%)	242 (26.4)	39 (4.3)	
1st WBC level (×10^9^/L), median (IQR)	16.6 (12.9–21.2)	18.6 (13.5–23.6)	0.2180
Time to 1st WBC level (hrs), median (IQR)	6.2 (2.6–9.7)	7.9 (3.2–16.5)	0.1042
Total 2nd WBC, n (%)	67 (7.3)	8 (0.9)	0.4262
2nd WBC level (×10^9^/L), median (IQR)	13.9 (9.9–17.0)	15.8 (10.5–23.7)	0.3851
Time to 2nd WBC level (hrs), median (IQR)	32.8 (20.6–45.3)	58.6 (41.7–77.7)	0.2234

CRP = C-reactive protein. EOS = early-onset sepsis. hrs = hours, IQR = interquartile range. PCT = procalcitonin. WBC = white blood cell count.

a 1 missing.

b 2 missing.

### Adverse events

There were 75 adverse events and 29 serious adverse events in the EOS calculator arm. In the categorical guideline arm 80 adverse events, and 44 serious adverse events occurred (Appendix, pp 8–10). Readmission for suspected early-onset sepsis occurred three times in the EOS calculator and two times in the categorical guideline arm. Any cultures obtained at readmission remained negative, and any symptoms resolved completely.

## Discussion

This study was conducted to assess whether the EOS calculator can safely reduce antibiotic exposure in newborns with suspected EOS, compared with a categorical guideline. This trial found that the use of the EOS calculator is non-inferior regarding adverse clinical outcomes and superior regarding the proportion of participants who received antibiotic therapy for suspected EOS within the first 24 h after birth. These results show that the EOS calculator is both a safe and effective tool for reducing unnecessary antibiotic exposure in newborns with suspected EOS. This supports the preceding observational implementation studies whilst mitigating concerns for missing severe disease.

Presence of harm criteria were expected to be either similarly distributed between both arms, or potentially more common in the EOS calculator arm, due to possibly delayed initiation of antibiotic therapy in that arm. Remarkably, the composite safety outcome occurred significantly less in the EOS calculator arm, with fewer initiations of respiratory support specifically notable. Several possible explanations were explored. Firstly, a direct beneficial effect of the EOS calculator use on respiratory health is unlikely; it guides clinical decision-making regarding the start of antibiotics, not respiratory management, and need for respiratory support is not a known side effect of intravenous antibiotics. Secondly, while randomisation aims to minimise bias and confounding, cluster randomised trials could signify a higher risk of imbalances compared to individually randomised trials. Specifically, respiratory distress was less common in the EOS calculator arm at baseline. Despite otherwise similar baseline characteristics, this may indicate that the clusters randomised for EOS calculator use may, by chance, admit fewer respiratory challenged newborns. Additionally, the absence of blinding may have invoked selection bias; the presence of respiratory distress is an important factor in categorical guidelines and may have prompted inclusion. However, it is also a factor in the EOS calculator (Appendix, p 2–3), and inclusion protocols were equal in both arms. Some local primary investigators suggested confounding by indication: when EOS calculator use demonstrates low risk, certain respiratory symptoms may be considered physiologic signs of the transition period and therefore managed with less respiratory support, compared with categorical guideline recommendation to start empiric antibiotics, with respiratory symptoms interpreted as progressive illness, warranting respiratory support. The lack of blinding makes this explanation plausible, but we have no data to further elucidate this hypothesis. Per-protocol analyses performed to account for post-randomisation factors, including crossover and non-adherence, did not reveal discrepant results. Correcting for respiratory distress at baseline changed the relative risk for presence of at least one harm criteria from 0.48 (95% CI 0.36–0.63) to 0.74 (95% CI 0.60–0.92). This implicates a partial confounding effect but still supports non-inferiority (and perhaps a potentially protective effect), indicating safety of the EOS calculator as a robust result. Future studies should consider respiratory distress as a stratification factor.

The use of the EOS calculator resulted in an absolute reduction in initiated antibiotic therapy for suspected EOS of 19.0%, which corresponds to a relative risk of 0.28 (95% CI 0.18–0.43). This reflects a stronger effect than the average relative risk of 0.56 reported by other implementation studies with similar eligibility criteria,^16^ and corresponds well to our preceding study of hypothetical EOS calculator use in a similar setting.^28^ The difference with other implementation studies is likely the result of a trial setting with better adherence to the comparator guideline than usual, resulting in a higher percentage of antibiotic prescription.^29^ When initiated, the average duration of therapy was found to be significantly longer in the EOS calculator arm, possibly explained by the fact that it allocates antibiotics to the proportion of newborns with the highest risk, making discontinuing antibiotics more challenging. However, this highlights a limitation of the EOS calculator, since risk assessment at initiation of antibiotic treatment does not inform on the need for continuation of antibiotics. During observation, we found more changes in neonatal clinical condition in the EOS calculator arm. This may reflect the emphasis on repeated clinical observation in this approach, although this remains speculation.

This randomised trial investigated the safety of EOS calculator usage, offering several strengths. The cluster-randomised design reduced contamination bias, which could occur if clusters would use the EOS calculator and categorical guideline simultaneously. It also allowed for comparison of two strategies for an urgent clinical decision shortly after birth, a time associated with elevated parental stress levels that preclude well-informed individual consent. In contrast to many EOS calculator studies limited to newborns of at least 35 weeks’ gestational age, this trial included those born at 34 weeks, extending generalisability. A follow-up period for all adverse events extending until day 14 after birth ensured safety regarding potential missed infections.

Limitations of this trial include a relatively small number of non-blinded clusters and inclusion depending on informed consent, which ensured feasibility and approval from medical-ethical committees but opens some possibilities for post-randomisation confounding. The true degree of clustering, though still minimal for non-inferiority, was higher than anticipated. However, analyses with cluster corrections showed almost identical results, suggesting limited impact. The trial was conducted in a single country with a single categorical guideline, limiting generalisability but ensuring a distinct and robust comparison. The use of a composite safety outcome may obscure the effects of individual harm criteria but allows for sufficient power given low incidences of individual outcomes. Our trial used minor modifications to the EOS calculator to adapt it to the Dutch setting. We used original coefficients to calculate EOS risk, whereas the EOS calculator was recently calibrated with updated coefficients.^30^ Unmodified or updated versions may be more appropriate for other settings or future updates. Any version of the EOS calculator should be used responsibly. Local regulations for decision support tools should be adhered to, and as with any EOS risk management strategy, clinical vigilance for early signs of EOS remains essential. EOS calculator use is only valid within 24 h after birth.

This trial demonstrated the safety and effectiveness of the EOS calculator by non-inferiority regarding adverse clinical outcomes and superiority in the proportion of participants who received antibiotic therapy in the Dutch neonatal population at risk for EOS. Building on preceding observational evidence, our findings may prompt revision of categorical guidelines to help protect newborns from harms of antibiotic overtreatment. Future research should stratify according to respiratory distress, consider new iterations of the EOS calculator, and aim for even further reduction of antibiotic overtreatment.

## Contributors

All authors contributed to data interpretation, critically reviewed drafts, and read and approved the final version of the manuscript. BW, SJ and MW verified the underlying data. All authors had full access to all the data in the trial and accept responsibility for the decision to submit for publication. Concept, design, draft writing, and funding acquisition: BW SJ SO DV LL RK AR MB FB NA. Statistical analysis: SJ MW. Patient recruitment and data collection: RC GT CL AK CLF JH HL MR NO IS. Supervision: FP NA.

## Data sharing statement

Data will be made available on request through a repository and shared after consent of the principal investigator and in accordance with local regulations.

## Declaration of interests

All authors declare no competing interests.

## Appendix A Supplementary data

EOS Calculator RCT–Appendix 2

Protocol–EOS Calculator RCT–v4.0 1mrt2022
