# Supplementary material for: Safety and effectiveness of the early-onset sepsis calculator to reduce antibiotic exposure in at-risk newborns: a cluster-randomised controlled trial
Source: eClinicalMedicine. 2025 Aug 12;87:103419. doi: 10.1016/j.eclinm.2025.103419 (PMC12359153; doi:10.1016/j.eclinm.2025.103419)
Supplement: EOS Calculator RCT–Appendix 2 [file mmc1.docx]

**SUPPLEMENTAL MATERIAL**

Table S1. Risk Factors Categorical Guidelines and EOS Calculator.....................................................................2

Figure S1. Decision Diagram for Dutch Categorical Guideline.............................................................................4

Figure S2. Decision Diagram for Early-Onset Sepsis Calculator...........................................................................5

Table S2. Duration of Harm Criteria......................................................................................................................6

Table S3. Protocol Deviations................................................................................................................................8

Table S4. Adverse Events.......................................................................................................................................7

References.............................................................................................................................................................11

**Table S1. Risk Factors Categorical Guidelines and EOS Calculator**

|  | **Dutch Categorical Guideline** | | **NICE Guideline** | | **EOS Calculator** | |
| --- | --- | --- | --- | --- | --- | --- |
|  | **Risk Factors** | **Class.** | **Risk Factors** | **Class.** | **Risk Factors** | **Class.** |
| **Maternal Risk Factors** | **Maternal parenteral antibiotic treatment with clinical symptoms of a sepsis during delivery or within 24 hours prior to or after delivery** | 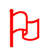 |  |  | Type of intrapartum antibiotics |  |
|  | **Suspected or confirmed infection in another baby in the case of a multiple pregnancy** | 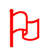 | **Suspected or confirmed infection in another baby in the case of a multiple pregnancy** | 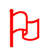 |  |  |
|  | Invasive GBS infection in a previous baby |  | Invasive GBS infection in a previous baby or maternal GBS colonisation, bacteriuria or infection in the current pregnancy |  | Maternal GBS status |  |
|  | Maternal GBS colonisation, bacteriuria, or infection in the current pregnancy |  |  |  |  |  |
|  | Pre-term birth following spontaneous labour before 37 weeks' gestation |  | Pre-term birth following spontaneous labour before 37 weeks' gestation |  | Gestational age |  |
|  | Confirmed prelabour rupture of membranes at term for more than 24 hours before the onset of labour |  | Confirmed prelabour rupture of membranes at term for more than 24 hours before the onset of labour |  | Duration rupture of membranes (hours) |  |
|  | Confirmed rupture of membranes for more than 18 hours before a pre-term birth |  | Confirmed rupture of membranes for more than 18 hours before a pre-term birth |  |  |  |
|  | Intrapartum fever higher than 38°C (regardless of epidural) or chorioamnionitis (suspected or proven) |  | Intrapartum fever higher than 38°C if there is suspected or confirmed bacterial infection |  | Highest maternal antepartum temperature |  |
| **Neonatal /Clinical Risk Factors** | **Onset respiratory distress >4 hours postpartum** | 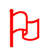 | **Apnoea (temporary stopping of breathing)** | 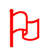 | Need for supplemental O_2_ > 2 hours to maintain oxygen saturations > 90% (outside of the delivery room) | Clinically Ill^*^ |
|  | **Seizures** | 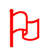 | **Seizures** | 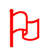 | Neonatal encephalopathy /Perinatal depression (defined as: seizures or Apgar score at 5 minutes <5). |  |
|  | **Signs of shock** | 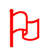 | **Signs of shock** | 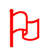 | Hemodynamic instability requiring vasoactive drugs |  |
|  | **Need for mechanical ventilation in a term neonate** | 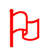 | **Need for mechanical ventilation** | 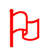 | Persistent need for CPAP / HFNC / mechanical ventilation (outside of the delivery room) |  |
|  | Need for mechanical ventilation in a preterm neonate |  |  |  |  |  |
|  | Need for cardiopulmonary resuscitation |  | **Need for cardiopulmonary resuscitation** | 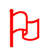 |  |  |
|  | Apnoea and bradycardia |  | Abnormal heart rate (bradycardia or tachycardia) |  | Persistent physiologic abnormality > 4 hours OR two or more physiologic abnormalities lasting for > 2 hours:  - Tachycardia (HR > 160)  - Tachypnoea (RR > 60)  - Respiratory distress (grunting, flaring, or retracting) not requiring supplemental O_2_  - Temperature instability (> 100.4˚F or < 97.5˚F) | Equivocal^†^ |
|  | Signs of respiratory distress (including grunting, recession, tachypnoea) |  | Signs of respiratory distress (including grunting, recession, tachypnoea) |  |  |  |
|  | Temperature abnormality (lower than 36°C or higher than 38°C) unexplained by environmental factors |  | Temperature abnormality (lower than 36°C or higher than 38°C) unexplained by environmental factors |  |  |  |
|  | Altered behaviour (for example, quiet [responsiveness], hypotonia) |  | Altered behaviour or responsiveness |  | No persistent physiologic abnormalities | Clinically Well |
|  |  |  | Altered muscle tone (for example, floppiness) |  |  |  |
|  | Feeding problems (for example, feed refusal, abdominal distention, vomiting) |  | Feed intolerance, including vomiting, excessive gastric aspirates and abdominal distension |  |  |  |
|  |  |  | Feeding difficulties (for example, feed refusal) |  |  |  |
|  | Hypoxia (for example, central cyanosis or reduced oxygen saturation level) |  | Hypoxia (for example, central cyanosis or reduced oxygen saturation level) |  |  |  |
|  | Signs of neonatal encephalopathy |  | Signs of neonatal encephalopathy |  |  |  |
|  | Persistent pulmonary hypertension of newborns |  | Persistent pulmonary hypertension of newborns |  |  |  |
|  | Local signs of infection (for example, on skin or eyes) |  | Jaundice within 24 hours of birth |  |  |  |
|  |  |  | Unexplained excessive bleeding, thrombocytopenia, or abnormal coagulation |  |  |  |
|  |  |  | Altered glucose homeostasis (hypoglycaemia or hyperglycaemia) |  |  |  |
|  |  |  | Metabolic acidosis (base deficit of 10 mmol/litre or greater) |  |  |  |

CPAP=continuous positive airway pressure. EOS=early-onset sepsis. GBS=Group B Streptococcus. HFNC=high flow nasal cannula. HR=heart rate. NICE=National Institute for Health and Care Excellence. O_2_=oxygen dioxide. RR=respiratory rate.

Table S2. Represents the risk factors used in the Dutch categorical guideline, the NICE guideline, and the variables used in the EOS calculator. Antibiotic treatment is advised, in both clinical guidelines, in case of presence of 1 red flag symptom or 2 non-red flag symptoms. ^*^One risk factor is needed to be categorised as ‘clinically ill’. ^†^Risk factors may be present intermittently to be categorised as ‘equivocal’.

Identify maternal risk factors and the clinical condition of the newborn for early

-

onset neonatal infection. If a red

flag is identified, immediate antibiotic treatment is indicated.

Perform a physical examination without delay if there are risk factors for early

-

onset sepsis.

•

red flag(s)

or

•

two or more risk

factors or clinical

symptoms

•

no clinical

symptoms

and

•

one risk factor

•

no risk factors

and

•

one clinical

symptom

•

no risk factors

and

•

no clinical

symptoms

Consider to observe the newborn for at least 12 hours

(monitor temperature and breathing at 1, 3, 6, 9 and 12

hours after birth).

I

ncreased

vigilance is

advised in

case

of

maternal fever (>38℃), premature rupture of

membranes (>24 hours), or GBS colonisation

Start antibiotic

treatment

Suspected infection

Consider to stop

antibiotic treatment

after 36

-

48 hours

Yes

No antibiotic

treatment. Normal

maternity care.

Reassure the family

members when the

neonate is discharged

Yes

GBS=Group B Streptococcus.

**Figure S1. Decision Diagram for Dutch Categorical Guideline**

The Dutch categorical guideline is an adaptation from the UK National Institute for Health and Care Excellence (NICE) 2012 guideline.^2,3^ Figure adapted from van der Weijden et al.^4^

Clinical illness

No

Yes

Adjusted EOS risk ≥3∙0/1000

Adjusted EOS risk 1∙0-3∙0/1000

No

**Monitor vital signs every 3**

**hrs for 24 hours after birth**

**Blood culture^⸸^**

EOS risk at birth <1∙0/1000

**Routinely monitor vital**

**signs^⸭^**

**Strongly consider administer**

**empirical**

**antibiotics**

**Vitals per**

**NICU**

No

Yes

No

Yes

Positive blood culture

or clinical illness

Adjusted EOS risk <3∙0/1000

•

Local incidence of early

-

onset sepsis^†^

•

Gestational age

•

Maximum maternal

intrapartum temperature

•

Duration of membrane

ruptures

•

Maternal GBS colonisation

status

•

Type and duration of

intrapartum antibiotics*

Adjusted EOS risk

Clinical Classification^‡^ with

associated likelihood ratios

•

Well

appearing

•

Equivocal exam

•

Clinical illness

EOS risk at birth

**Obtain blood culture and**

**administer empirical**

**antibiotics.**

**Vitals per NICU**

Yes

Adjusted EOS risk ≥3∙0/1000

**Monitor vital signs every 3**

**hrs for 24 hours after birth**

Clinical illness

Clinical illness

GBS=Group B Streptococcus. NICU=neonatal intensive care unit.

**Figure S2. Decision Diagram for Early-Onset S**[**epsis**](https://www.sciencedirect.com/topics/pharmacology-toxicology-and-pharmaceutical-science/sepsis)**Calculator**

Figure partly adapted from Benitz and Achten.^5^ The dotted arrow indicates suggested progression.^*^ Antibiotic classification used as defined by Kuzniewicz and colleagues,^6^ with addition of amoxicillin/clavulanic acid specified as broad-spectrum antibiotic prophylaxis. ^‡^Clinical signs and classification as defined by Kuzniewicz and colleagues. ^6^ ^†^Incidence of 0∙6/1000 live births was used. ^⸭^All hospitals followed a 24 hour observation period with vital signs every 3 hours. ^⸸^Blood culture was not obtained in this trial

**Table S2. Duration of Harm Criteria**

| **Non-inferiority & Superiority** | **Categorical Guideline N=915** | **EOS Calculator**  **N=915** | **RR (95% CI)** | **P-value** |
| --- | --- | --- | --- | --- |
| **Co-primary non-inferiority outcome** |  |  |  |  |
| *Harm criteria* |  |  |  |  |
| Need for respiratory support, n (%) | 133 (14·5) | 62 (6·8) | 0·47 (0·39–0·56) | 0·0000 |
| Invasive/Mechanical ventilation, n (%) | 7 (0·8) | 4 (0·4) |  |  |
| Duration in hours, median (IQR) | 22·0 (14·0–30·0) | 0·1 (0·0–0·1) |  | 0·0581 |
| CPAP, n (%) | 117 (12·8) | 63 (6·9) |  |  |
| Duration in hours, median (IQR) | 10 (4·0–21·0)^a^ | 8·0 (2·0–16·0) |  | 0·1234^a^ |
| High flow (>2L/min.), n (%) | 31 (3·4) | 9 (1·0) |  |  |
| Duration in hours, median (IQR) | 29·5 (16·0–54·0) | 51·0 (28·5–95·0) |  | 0·2560 |
| Low flow (≤2L/min.), n (%) | 33 (3·6) | 15 (1·6) |  |  |
| Duration in hours, median (IQR) | 29·0 (10·0–49·0) | 24·0 (8·5–49·0) |  | 0·3502 |
| Total duration of respiratory support in hours, median (IQR) | 15·8 (5·3–38·8)^a^ | 9·4 (2·5–28·0) |  | 0·1195^a^ |
| Need for supplemental oxygen, n (%) | 93 (10·2) | 55 (6·0) | 0·59 (0·41–0·86) | 0·0059 |
| Duration of supplemental oxygen in hours, median (IQR) | 2·0 (0·0–9·8)^a^ | 1·0 (0·0–8·0)^a^ |  | 0·3387^b^ |
| Duration >30% oxygen in hours, median (IQR) | 2·0 (0·0–5·7)^a^ | 1·0 (0·0–8·0)^a^ |  | 0·2132^b^ |
| Duration >50% oxygen in hours, median (IQR) | 0·0 (0·0–1·0)^a^ | 0·0 (0·0–1·0) |  | 0·9519^a^ |

CPAP=continuous positive airway pressure. EOS=early-onset sepsis. IQR=interquartile range. RR=relative risk.

^a^1 missing, ^b^2 missing.

**Table S3.** **Protocol Deviations**

| **Protocol Deviations** | **Categorical Guideline** **N=915** | **EOS Calculator**  **N=915** |
| --- | --- | --- |
| **Excluded from per protocol population, n (%)** | 114 (12·5) | 96 (10·5) |
| ***Cross-over from categorical guideline to EOS calculator or vice versa, n (%)*** | 0 (0) | 14 (1·5) |
| due to missing data, n (%) | 0 (0) | 5 (0·5) |
| reason unknown, n (%) | 0 (0) | 9 (1·0) |
| ***Non-adherence to treatment advice, n (%)*** | 114 (12·5) | 49 (5·4) |
| no AB given (when treatment was advised), n (%) | 62 (6·8) | 9 (1·0) |
| AB given (when treatment was not advised), n (%) | 52 (5·7) | 40 (4·4) |
| ***Discrepant data used*, n (%)*** | 2 (0·2) | 36 (3·9) |
|  |  |  |
| **Not excluded from per protocol population, n (%)** | 8 (0·9) | 41 (4·5) |
| No first evaluation, n (%) | 0 (0) | 2 (0·2) |
| First evaluation during first 30 minutes, n (%) | 0 (0) | 2 (0·2) |
| No vitals every 3 hours, n (%) | 0 (0) | 1 (0·1) |
| Less observation duration, n (%) | 0 (0) | 1 (0·1) |
| Maternal data consent denied, n (%) | 2 (0·2) | 2 (0·2) |
| EOS calculator result not saved, n (%) | 0 (0) | 28 (3·1) |
| Other, n (%) | 6 (0·7) | 5 (0·5) |
| Participants with one or more protocol violations, n (%) | 121 (13·2) | 134 (14·6) |

AB=antibiotics. EOS=early-onset sepsis. *Data important for EOS risk management but inconsistent with the electronic health record. For example, ‘Broad-spectrum antibiotics > 4 hours prior to birth’ was used as input for the ‘Type of intrapartum antibiotics’ variable of the EOS calculator, while no administration of intrapartum antibiotics was recorded in the electronic health record.

**Table S4. Adverse Events**

| **Adverse Events** | **Categorical Guideline** **N=915** | **EOS Calculator**  **N=915** |
| --- | --- | --- |
| **Total of (serious) adverse events, n (%)** | 124 (13·6) | 104 (11·4) |
| **Adverse events, n (%)** | 80 (8·7) | 75 (8·2) |
| Abnormal laboratory results, n (%) | 9 (1·0) | 15 (1·6) |
| Abnormal test results, n (%) | 6 (0·7) | 1 (0·1) |
| Probable life-threatening event, n (%) | 0 (0) | 1 (0·1) |
| Extended hospitalisation, n (%) | 2 (0·2) | 8 (0·9) |
| GP visit, n (%) | 13 (1·4) | 12 (1·3) |
| Conjunctivitis, n (%) | 2 (0·2) | 1 (0·1) |
| Thrush, n (%) | 5 (0·5) | 2 (0·2) |
| Upper respiratory infection, n (%) | 1 (0·1) | 1 (0·1) |
| Vaginal yeast infection, n (%) | 1 (0·1) | 0 (0) |
| Intertrigo, n (%) | 1 (0·1) | 0 (0) |
| Tachypnoea, n (%) | 1 (0·1) | 0 (0) |
| Oral candidiasis, n (%) | 0 (0) | 1 (0·1) |
| Abdominal cramps, n (%) | 1 (0·1) | 0 (0) |
| Constipation, n (%) | 0 (0) | 1 (0·1) |
| Impetigo, n (%) | 0 (0) | 1 (0·1) |
| Feeding difficulties, n (%) | 1 (0·1) | 0 (0) |
| Regular checkup, n (%) | 0 (0) | 1 (0·1) |
| Cow's milk protein allergy, n (%) | 0 (0) | 1 (0·1) |
| Unknown reason, n (%) | 0 (0) | 3 (0·3) |
| Other doctor visit, n (%) | 4 (0·4) | 8 (0·9) |
| Prematurity, n (%) | 1 (0·1) | 0 (0) |
| Suspected hyperbilirubinemia, n (%) | 2 (0·2) | 4 (0·4) |
| Well Baby Clinic, n (%) | 1 (0·1) | 1 (0·1) |
| Unknown reason, n (%) | 0 (0) | 3 (0·3) |
| ER visit, without readmission, n (%) | 5 (0·5) | 8 (0·9) |
| Fever, n (%) | 1 (0·1) | 0 (0) |
| Weight loss, n (%) | 1 (0·1) | 0 (0) |
| Hyperbilirubinemia, n (%) | 2 (0·2) | 2 (0·2) |
| Bilious vomiting, n (%) | 0 (0) | 1 (0·1) |
| Lethargy and feeding difficulty, n (%) | 0 (0) | 1 (0·1) |
| Inconsolable crying, n (%) | 0 (0) | 1 (0·1) |
| Malaise, n (%) | 0 (0) | 1 (0·1) |
| Vomiting and diarrhoea, n (%) | 0 (0) | 1 (0·1) |
| Unknown reason, n (%) | 1 (0·1) | 1 (0·1) |
| ER visit, unknown course, n (%) | 0 (0) | 1 (0·1) |
| NICU referral, n (%) | 22 (2·4) | 2 (0·2) |
| Respiratory support local policy, n (%) | 12 (1·3) | 0 (0) |
| CPAP, n (%) | 8 (0·9) | 0 (0) |
| CPAP and HFNC, n (%) | 1 (0·1) | 0 (0) |
| HFNC, n (%) | 2 (0·2) | 0 (0) |
| CPAP and hypoglycaemia, n (%) | 1 (0·1) | 0 (0) |
| Respiratory support for prematurity, n (%) | 10 (1·1) | 2 (0·2) |
| Conjunctivitis, n (%) | 2 (0·2) | 1 (0·1) |
| Erb’s palsy, n (%) | 1 (0·1) | 0 (0) |
| Upper respiratory infection, n (%) | 2 (0·2) | 1 (0·1) |
| Antibiotics missed once, n (%) | 1 (0·1) | 0 (0) |
| Frenotomy, n (%) | 3 (0·3) | 1 (0·1) |
| Hyperextension, hypotonia, distended abdomen, n (%) | 1 (0·1) | 0 (0) |
| Echocardiogram (maternal cutaneous lupus), n (%) | 1 (0·1) | 0 (0) |
| Reflux, n (%) | 1 (0·1) | 0 (0) |
| Dermatitis, n (%) | 2 (0·2) | 0 (0) |
| Perianal dermatomycosis, n (%) | 1 (0·1) | 0 (0) |
| Vomiting, n (%) | 1 (0·1) | 0 (0) |
| Subfebrile temperature, n (%) | 1 (0·1) | 0 (0) |
| Decreased alertness, n (%) | 1 (0·1) | 0 (0) |
| Polycythaemia, n (%) | 1 (0·1) | 0 (0) |
| Hypothermia, n (%) | 0 (0) | 2 (0·2) |
| Inconsolable crying, n (%) | 0 (0) | 1 (0·1) |
| Swelling of the left thigh, n (%) | 0 (0) | 1 (0·1) |
| Cephalohematoma, n (%) | 0 (0) | 1 (0·1) |
| Hypotonia, bradypnea, persistent extra oxygen requirement, n (%) | 0 (0) | 1 (0·1) |
| Undescended testis, n (%) | 0 (0) | 1 (0·1) |
| Epstein’s pearls on penis, n (%) | 0 (0) | 1 (0·1) |
| Suspected clavicle fracture, n (%) | 0 (0) | 1 (0·1) |
| Unknown, n (%) | 0 (0) | 1 (0·1) |
| Skin lesion, n (%) | 0 (0) | 1 (0·1) |
| Bacteriuria without treatment, n (%) | 0 (0) | 1 (0·1) |
| Blood sample without treatment, n (%) | 0 (0) | 1 (0·1) |
| Suspected ventricular extrasystoles, n (%) | 0 (0) | 1 (0·1) |
| Sickle cell, n (%) | 0 (0) | 1 (0·1) |
| Feeding difficulties, n (%) | 0 (0) | 1 (0·1) |
| **Serious adverse events, n (%)** | 44 (4·8) | 29 (3·2) |
| NICU referral, n (%) | 10 (1·1) | 3 (0·3) |
| Sepsis treatment, n (%) | 5 (0·5) | 0 (0) |
| Blood aspiration, n (%) | 1 (0·1) | 0 (0) |
| Pneumothorax, n (%) | 1 (0·1) | 1 (0·1) |
| PPHN, n (%) | 1 (0·1) | 0 (0) |
| Suspected volvulus, n (%) | 1 (0·1) | 0 (0) |
| Stridor, n (%) | 0 (0) | 1 (0·1) |
| Middle cerebral artery infarct, n (%) | 0 (0) | 1 (0·1) |
| Ventricular extrasystoles, n (%) | 1 (0·1) | 0 (0) |
| Readmission, n (%) | 34 (3·7) | 26 (2·8) |
| Suspected EOS, n (%) | 2 (0·2) | 3 (0·3) |
| Confirmed EOS, n (%) | 0 (0) | 0 (0) |
| Suspected meningitis/LOS, n (%) | 2 (0·2) | 5 (0·5) |
| Confirmed meningitis/LOS, n (%) | 0 (0) | 3 (0·3) |
| Suspected viral infection, n (%) | 2 (0·2) | 1 (0·1) |
| Hyperbilirubinemia, n (%) | 15 (1·6) | 7 (0·8) |
| Hyperbilirubinemia and weight loss, n (%) | 1 (0·1) | 2 (0·2) |
| Weight loss, n (%) | 1 (0·1) | 3 (0·3) |
| Reflux, n (%) | 0 (0) | 2 (0·2) |
| Feeding difficulties, n (%) | 4 (0·4) | 0 (0) |
| Hypothermia, n (%) | 1 (0·1) | 1 (0·1) |
| Apnoea and hypotonia, n (%) | 1 (0·1) | 0 (0) |
| Myoclonic seizures, n (%) | 1 (0·1) | 0 (0) |
| Laryngomalacia, n (%) | 1 (0·1) | 0 (0) |
| Cephalhematoma, n (%) | 1 (0·1) | 0 (0) |
| Fall from height, n (%) | 0 (0) | 1 (0·1) |
| Cerebral haemorrhage, n (%) | 0 (0) | 1 (0·1) |
| Unexplained hematoma, n (%) | 1 (0·1) | 0 (0) |
| Inconsolable crying, n (%) | 1 (0·1) | 0 (0) |
|  |  |  |
| Participants with one or more adverse events, n (%) | 120 (13·1) | 103 (11·3) |

CPAP=continuous positive airway pressure. EOS=early-onset sepsis. ER=emergency room. GP=general practitioner. HFNC=high flow nasal cannula. LOS=late-onset sepsis. NICU=neonatal intensive care unit. PICU=pediatric intensive care unit. PPHN=persistent pulmonary hypertension of the newborn.

**References**

1. Campbell MK, Piaggio G, Elbourne DR, Altman DG, Group C. Consort 2010 statement: extension to cluster randomised trials. *BMJ* 2012; **345**: e5661.

2. Nederlandse Vereniging voor Kindergeneeskunde. Preventie en behandeling van early-onset neonatale infecties (Adaptatie van de NICE-richtlijn). Utrecht, Netherlands: Nederlandse Vereniging voor Kindergeneeskunde (NVK), 2017.

3. National Institute for Health and Care Excellence (NICE). Neonatal infection (early onset): antibiotics for prevention and treatment (CG149). London, UK: National Institute for Health and Care Excellence (NICE), 2012.

4. van der Weijden BM, van der Weide MC, Plotz FB, Achten NB. Evaluating safety and effectiveness of the early-onset sepsis calculator to reduce antibiotic exposure in Dutch at-risk newborns: a protocol for a cluster randomised controlled trial. *BMJ Open* 2023; **13**(2): e069253.

5. Achten NB, Dorigo-Zetsma JW, van der Linden PD, van Brakel M, Plötz FB. Sepsis calculator implementation reduces empiric antibiotics for suspected early-onset sepsis. *European Journal of Pediatrics* 2018; **177**(5): 741-6.

6. Kuzniewicz MW, Walsh EM, Li S, Fischer A, Escobar GJ. Development and implementation of an early-onset sepsis calculator to guide antibiotic management in late preterm and term neonates. *Joint Commission Journal on Quality and Patient Safety* 2016; **42**(5): 232-9.
