# Supplementary material for: Safety and effectiveness of the early-onset sepsis calculator to reduce antibiotic exposure in at-risk newborns: a cluster-randomised controlled trial
Source: eClinicalMedicine. 2025 Aug 12;87:103419. doi: 10.1016/j.eclinm.2025.103419 (PMC12359153; doi:10.1016/j.eclinm.2025.103419)
Supplement: Protocol–EOS Calculator RCT–v4.0 1mrt2022 [file mmc2.pdf]

Safely reduce newborn antibiotic exposure with the early-onset sepsis calculator: a cluster randomized study (EOS Calculator RCT)

March 2022

PROTOCOL TITLE: Safely reduce newborn antibiotic exposure with the early-onset sepsis calculator: a cluster randomized study (EOS Calculator RCT)

**Protocol ID**	N/A
**Short Title**	EOS Calculator RCT
**EudraCT Number**	N/A
**Version**	4.0
**Date**	March 1st, 2022
**Coordinating Investigators**	Drs. Bo M. van der Weijden<br>Tergooi Ziekenhuis, Rijksstraatweg 1, 1261 AN Blaricum<br><br>Dr. Niek B. Achten<br>Amphia ziekenhuis, Molengracht 21<br>4818 CK Breda
**Principal Investigator**	Prof. dr. Frans B. Plötz, paediatrician<br>Tergooi Ziekenhuis, Rijksstraatweg 1, 1261 AN Blaricum<br>Amsterdam UMC, Meibergdreef 9, 1105 AZ Amsterdam
**Subsidising Party**	De Nederlandse Vereniging voor Kindergeneeskunde (NVK) middels een bijdrage van het Prins Bernard Cultuurfonds aan de NVK.
**Independent Expert**	Dr. Wieger P. Voskuijl,<br>Amsterdam UMC, Meibergdreef 9, 1105 AZ Amsterdam<br>w.p.voskuijl@amsterdamumc.nl

TABLE OF CONTENTS

1. INTRODUCTION AND RATIONALE 8
2. OBJECTIVES 11
3. STUDY DESIGN 12
4. STUDY POPULATION 15
   4.1 Population (base) 16
   4.2 Inclusion criteria 16
   4.3 Exclusion criteria 16
   4.4 Sample size calculation 16
5. INVESTIGATIONAL PRODUCT 18
   5.1 Name and description of investigational product 18
   5.2 Summary of findings from non-clinical studies 19
   5.3 Summary of findings from clinical studies 19
   5.4 Summary of known and potential risks and benefits 20
6. METHODS 21
   6.1 Study parameters/endpoints 21
      6.1.1 Main study parameter/endpoint 21
      6.1.2 Secondary study parameters/endpoints 21
   6.2 Randomization, blinding and treatment allocation 22
   6.3 Study procedures 22
   6.4 Withdrawal of individual subjects 23
   6.5 Replacement of individual subjects after withdrawal 24
   6.6 Follow-up of subjects withdrawn from treatment 24
      6.6.1 Premature termination of the study 24
7. SAFETY REPORTING 25
   7.1 Temporary halt for reasons of subject safety 25
   7.2 AEs and SAEs 25
      7.2.1 Adverse events (AEs) 25
      7.2.2 Serious adverse events (SAEs) 26
   7.3 Annual safety report 27
   7.4 Follow-up of (serious) adverse events 27
   7.5 Data Safety Monitoring Board (DSMB) / Safety Committee 27
8. STATISTICAL ANALYSIS 28
   8.1 Primary study parameters 28
   8.2 Secondary study parameters 28
      8.2.1 Quality of life 28
   8.3 Interim analysis 28
9. ETHICAL CONSIDERATIONS 29
   9.1 Regulation statement 29
   9.2 Recruitment and consent 29
   9.3 Objection by minors or incapacitated subjects 31
   9.4 Benefits and risks assessment, group relatedness 31
   9.5 Compensation for injury 32

10. ADMINISTRATIVE ASPECTS, MONITORING AND PUBLICATION 33
10.1 Handling and storage of data and documents 33
10.2 Monitoring and Quality Assurance 34
10.3 Amendments 34
10.4 Annual progress report 35
10.5 Temporary halt and (prematurely) end of study report 35
10.6 Public disclosure and publication policy 35
11. STRUCTURED RISK ANALYSIS 36
11.1 Potential issues of concern 36
11.2 Synthesis 37
12. REFERENCES 38

LIST OF ABBREVIATIONS AND RELEVANT DEFINITIONS

**ABR**	General Assessment and Registration form (ABR form), the application form that is required for submission to the accredited Ethics Committee; in Dutch: Algemeen Beoordelings- en Registratieformulier (ABR-formulier)
**AE**	Adverse Event
**CA**	Competent Authority
**CCMO**	Central Committee on Research Involving Human Subjects; in Dutch: Centrale Commissie Mensgebonden Onderzoek
**CV**	Curriculum Vitae
**DSMB**	Data Safety Monitoring Board
**EOS**	Early-Onset Sepsis
**EU**	European Union
**EudraCT**	European drug regulatory affairs Clinical Trials
**GCP**	Good Clinical Practice
**GDPR**	General Data Protection Regulation; in Dutch: Algemene Verordening Gegevensbescherming (AVG)
**IC**	Informed Consent
**METC**	Medical Research Ethics Committee (MREC); in Dutch: Medisch Ethische ToetsingsCommissie (METC)
**NTR**	Netherlands Trial Register (https://www.trialregister.nl/)
**NVK**	Nederlandse Vereniging voor Kindergeneeskunde
**(S)AE**	(Serious) Adverse Event
**Sponsor**	The sponsor is the party that commissions the organisation or performance of the research, for example a pharmaceutical company, academic hospital, scientific organisation or investigator. A party that provides funding for a study but does not commission it is not regarded as the sponsor, but referred to as a subsidising party.
**UAVG**	Dutch Act on Implementation of the General Data Protection Regulation; in Dutch: Uitvoeringswet AVG
**WMO**	Medical Research Involving Human Subjects Act; in Dutch: Wet Medisch-wetenschappelijk Onderzoek met mensen

SUMMARY

Rationale: Newborns are at risk for early-onset, within 72 hours after birth, sepsis (EOS). The incidence of proven EOS is 0.5-2.0 per 1000 live births. For the Netherlands, based on 170,000 births per year, this means approximately 85-340 newborns. However, approximately 5% of late preterm and term newborns are given antibiotics in compliance with current Dutch guideline, which is equivalent to 8,500 newborns per year. An alternative is the CE certified EOS calculator application, which calculates an individual risk of EOS for each newborn with treatment advice.

Objective: To investigate whether the use of the EOS calculator safely reduces antibiotic exposure in newborns with suspected EOS compared to the current Dutch guidelines.

Study design: A cluster-randomized study design (randomization at hospital level) with 10 hospitals.

Study population: Newborns in the first 24 hours after birth, at a gestational age of ≥34 weeks, with at least one risk factor or symptom consistent with EOS.

Intervention: Randomization will take place at cluster level for either the EOS calculator or antibiotics administration according to the Dutch guideline.

Main study parameters/endpoints: *Co-primary superiority outcome:* The proportion of patients starting antibiotic therapy started for suspected and, or proven EOS in the first 24 hours after birth. *Co-primary non-inferiority outcome:* The presence of one or more of four predefined safety criteria, namely 1) the need for any respiratory support, and/or 2) the need for an intravascular fluid bolus for hemodynamic instability due to sepsis, and/or 3) referral to a Neonatal Intensive Care Unit for sepsis treatment, and/or 4) proven EOS.

Secondary endpoints: The total duration of antibiotic therapy; The percentage antibiotic therapy started for suspected and, or proven EOS if symptoms started between 24-72 hours after birth; Quality of life: To get an impression of the impact of (suspected) EOS on parents/guardians and their child, parents/guardians will be asked to fill in a questionnaire on day 14.

Nature and extent of the burden and risks associated with participation, benefit, and group relatedness: This study will have negligible risks and minimal burden.

Burden: -Newborns who receive antibiotic therapy: An intravenous cannula will be inserted for antibiotic treatment and blood sampling. A second blood sample by venipuncture will be collected after 24 hours. This is part of standard medical treatment. One questionnaire will be sent to the parents/guardians to fulfill on day 14, which is extra for this study. The questionnaire includes a minimum of 22 questions, which may expand up to 32 questions (since some questions are dependent of other questions). It will take about 10-15 minutes to complete. -Newborns who do not receive antibiotic therapy: No blood samples taken. Clinical observation for at least 12-24 hours. This is part of standard medical treatment. **Risks:** -The risks

associated with participation are very low because newborns will be closely observed when no antibiotics will be prescribed. **Benefits:** -More specific risk analysis. Not all subjects will benefit personally from study participation. When no antibiotics will be prescribed: no separation of mother and child, no painful procedures, and a shorter hospital stay (including fewer costs), and no unnecessary exposure to antibiotics. **Group relatedness:** This study can only be performed with this specific group of patients, as it will provide age-specific and risk factor-specific data that cannot be obtained otherwise.

1. INTRODUCTION AND RATIONALE

Newborns are at risk for early-onset sepsis (EOS) (1,2). A (suspected) EOS is defined as a (suspected) infection that develops within 72 hours after birth. The incidence of confirmed EOS is 0.5-2.0 per 1000 live births. For the Netherlands, this translates to approximately 85-340 EOS cases yearly.

Suspicion of EOS is often based by doctors on risk factors and nonspecific clinical symptoms, such as maternal fever during labor or rapid breathing in the newborn. The classic infection parameters in the blood of newborns are of little to no value to rule out an infection at the time of presentation (3). The combination of the difficulty to recognize EOS in time and the risk of serious consequences of EOS not treated timely has resulted in a low threshold for the start of empirical antibiotic treatment. This has led to significant overtreatment; it is estimated that in the Netherlands about 5% of all newborns receive antibiotic therapy for suspected EOS, equivalent to 8,500 newborns per year (4–6), compared to the estimated 85-340 of confirmed EOS cases. Adverse consequences of antibiotic overtreatment are increasingly recognized (4). In the short term, it leads to separation of parents and newborn, invasive painful procedures (with possible long-term consequences for brain development), and unnecessary hospitalization with associated costs. In the longer term, there are significant disadvantages for the microbiome and immune system, found to be associated with obesity (7), eczema, asthma (8,9), and diabetes later in life (10). In addition, at the population level, overuse of antibiotics leads to resistant infections that are difficult to treat.

The current standard of care is the NVK guideline “Prevention and treatment of early-onset neonatal infections” (11). The aim of this guideline is uniform, evidence-based, diagnosis and treatment of early-onset neonatal infections, including careful use of antibiotics. The NVK guideline is an adaptation of the 2012 version of the National Institute for Health and Care Excellence (NICE) guideline "Antibiotics for early-onset neonatal infection: antibiotics for the prevention and treatment of early-onset neonatal infection" (12).

The NVK guideline uses risk factors and symptoms to categorically indicate which newborns need antibiotics (11). Evaluating its use in 9 Dutch hospitals, we found limited adherence, especially when antibiotics are recommended by the guideline (13). This limited adherence indicates that it is desirable to amend the current guideline or that the use of an alternative method should be further investigated.

A new method for determining which newborns need antibiotics is the “EOS calculator” (14,15). Developed in the United States, this method uses a combination of detailed information about risk factors and the presence of specific symptoms to calculate an individual risk of EOS for each newborn. The EOS calculator is an algorithm that divides newborns into risk groups with concrete treatment advice (antibiotics, extra observation, normal care). The EOS calculator calculates the risk for an individual newborn using 5 maternal risk factors (gestational age, duration of rupture of membranes, highest intrapartum maternal temperature, use of intrapartum antibiotics, maternal Group B Streptococcus (GBS) colonization status) combined with the newborn’s clinical condition after birth. These data are objective and easy to retrieve, without additional actions.

The EOS calculator has been developed and validated using data of over 600,000 newborns born with a gestational age of 34 weeks or more. A subsequent large-scale implementation study included 204,485 newborns and showed a decrease in antibiotic use from 5.0% to 2.6% (16). EOS incidence and readmissions for EOS did not change after implementation, nor were any other adverse effects observed. The EOS calculator has now been evaluated in more than 20 studies, of which 8 with actual implementation. Meta-analysis shows up to an average of 44% less antibiotic use, without indications of negative consequences (17). Implementation in Tergooi hospital yielded comparable results (18). There are no known implementation studies in which EOS calculator use was not effective or proven unsafe.

For the Dutch situation, equipoise between the current guideline and the EOS calculator can be established given balance of risk for both over- and undertreatment of EOS for both strategies: the current guideline is likely to result in more overtreatment with adverse consequences, but potentially carries a smaller risk for treatment delay (although it is unclear to what clinical significance). A large body of evidence now supports the safety of the EOS calculator approach, but validation outside of the North-American setting is limited, especially for safety outcomes. Equipoise between both strategies is confirmed by the recently updated version of the NICE guideline (the NVK guideline was based on the 2012 version), which includes use of the ‘EOS calculator’, as a valid alternative approach to allocate antibiotics to newborns.(19) Equipoise can also be inferred from recommendations of the American Academy of Pediatrics, which considers both a risk-factor based approach and the EOS calculator as valid strategies. We compared the EOS calculator to the NVK guideline in a post-hoc analysis of our multicenter study (20), and found that the EOS calculator recommended significantly fewer antibiotics than the NVK guideline. This result is in line with the results of previous international research showing that the EOS calculator is proven to be effective and safe for reducing antibiotic overtreatment (17,21).

A prospective cluster-randomized Dutch validation study of the EOS calculator is needed before complete adoption of the EOS calculator, because of the following reasons:

1) Multicenter validation of actual EOS calculator use has not been performed in the Netherlands.

2) To date, no randomized prospective study of EOS calculator use with safety as an outcome measure has been performed. Specifically, there are no studies that take into account the potential effects of a treatment delay due to EOS calculator use. This especially holds true for cases that are clinically suspect for EOS, but not culture-confirmed. The study here proposed by us is, to the best of our knowledge, the first.

This protocol describes a cluster-randomized trial, which is the logical next step to our previous research (18,22–28). The study aims to investigate whether the use of the EOS calculator safely reduces antibiotic exposure in newborns with suspected EOS compared to the current Dutch guideline.

2. OBJECTIVES

The primary objectives of this study are:

1. To investigate whether the use of the EOS calculator safely reduces antibiotic exposure in newborns with suspected EOS in the first 24 hours after birth.
2. To investigate the presence of one or more of the following four predefined safety criteria, namely 1) the need for any respiratory support, and/or 2) the need for an intravascular fluid bolus for hemodynamic instability due to sepsis, and/or 3) referral to a Neonatal Intensive Care Unit for sepsis treatment, and/or 4) proven EOS.

Secondary objectives of the study are:

1. To investigate if the use of the EOS calculator decreases the total duration of antibiotic therapy in newborns with suspected EOS.
2. To investigate if the use of the EOS calculator decreases the percentage of antibiotic therapy started for suspected and, or proven EOS if symptoms started between 24-72 hours after birth.
3. To study the impact of (suspected) EOS on parents/guardians.

3. STUDY DESIGN

Design: We will conduct a prospective, cluster-randomized trial. We aim to include 1830 newborns from 10 hospitals in the Netherlands during an 18-month period. Cluster-randomization happens at the hospital level. A cluster is finished when a total of 183 participants are included into the cluster. Once finished, a hospital cannot be randomized again. Participating hospitals provide care up to Level II special care for stable or moderately ill newborns, with annual birth rates between 1200 and 4000 births per year, and all use the Dutch national guidelines to guide antibiotic use in newborns at-risk for EOS.

This cluster randomized design can be used when randomization at individual level is difficult and/or creates possibility for bias. The cluster-randomized study design matches our study objectives perfectly because time for randomization is very restricted due to the immediate need to start antibiotic therapy in some newborns. Furthermore, the clustered approach prevents confusion and contamination between the different protocols being compared, thereby limiting bias. After randomization, the assigned protocol is considered standard care for newborns with suspected EOS within 0-24 hours after birth during the study period in that hospital. This means that the NVK guidelines or the EOS calculator (depending on randomization) will be used for all newborns at risk for EOS (within 0-24 hours after birth) in the particular hospital, independent of study participation. It is noteworthy that every attending physician always has the option to choose for clinical management according to another protocol instead of the assigned protocol.

Newborns who do not meet the inclusion criteria, and develop clinical symptoms (suspected for EOS) >24 hours after birth, the NVK guidelines will be used since the EOS calculator is only validated for use in the first 24 hours after birth. If newborns are eligible for inclusion and attending physicians opt for clinical management not according to the advice of the assigned study protocol, this will be recorded with deviations being noted with accompanying justification in both the electronic health record and the electronic Case Report Form (eCRF). After informed consent is obtained data will be collected and used for analysis, and follow-up data collection will ensue.

In order to meet requirements of the European Regulations for Medical Devices, the EOS calculator has been developed as a smartphone application specifically for this study, and CE marking has successfully been obtained (see chapter 5). Only in the five hospitals randomized for the EOS calculator this application will be available for all physicians who have a treatment relationship with newborns at risk for EOS.

Equality: We use the same strategy to include and observe participating newborns to ensure that the control group and the intervention group are equally recruited and monitored. Each newborn in participating hospitals is evaluated for elevated EOS risk. In case one or more criteria for elevated maternal EOS risk or neonatal clinical signs of EOS are met (in accordance with the NVK guideline, Table 1), clinical evaluation of the newborn by a pediatric resident or pediatrician will take place within 4 hours after birth. After concluding eligibility, the advice of either the NVK guideline or the EOS calculator (depending on randomization) to observe or to start antibiotic therapy is followed. We maintain an observation period of at least 12-24 hours for the NVK guideline-group and at least 24 hours for the EOS calculator-group. These observation periods are in accordance with the advice of both guidelines. Observation in both groups will consist of controls of vital parameters (heart rate, respiratory rate, and temperature) every 3 hours by the nurse, and a minimum of two physical examinations (within 4 hours after birth and before discharge) by the physician.

Usual care NVK guideline (control group): The NVK guidelines use eight maternal and fifteen neonatal risk factors, each categorized as either red flag or non-red flag (Table 1, see next page). These criteria guide clinicians on the management in case of suspected EOS. Briefly, antibiotic treatment is recommended in the presence of at least one red flag and, or, two or more non-red flags. Before the start of antibiotic treatment, the guidelines advise to take a blood culture, whereas a C-reactive protein (CRP) level can be considered. They advise to continue antibiotic treatment for at least 36-48 hours, which is based on the time to positivity of blood cultures. The guidelines advise to repeat a CRP after 24-36 hours after the start of antibiotic treatment. Discontinuation of antibiotic treatment after 36 hours can be considered in case of a negative blood culture and when the initial clinical suspicion of infection was not strong, the newborn’s clinical condition is reassuring with no clinical indicators of possible infection, and CRP concentrations are repeatedly below <10 mg/L.

An observation period of at least 12 hours is recommended in the presence of one non-red flag (this could be a maternal risk factor or a clinical symptom of the neonate). For this study we maintain an observation period of at least 12-24 hours. Antibiotic treatment is recommended when an infection is suspected during this observation. Newborns will be discharged from the hospital if there are no maternal risk factors, and the newborn is in good clinical condition and the gestational age is more than 36 weeks. If the guidelines recommend an observation period, the newborn is discharged after repeating physical examination that conclude that the newborn is in good clinical condition. In case antibiotic treatment is started, discharge depends on the duration of treatment and the clinical course. At discharge,

parents/guardians receive information when to contact the hospital in case of signs of infection within the first 14 days of life.

Table 1. Maternal and Neonatal Risk Factors for EOS in the Dutch guidelines

**Maternal risk factors**	**Neonatal risk factors**
**Red flags**	
Parenteral antibiotic treatment given to the woman for confirmed or suspected invasive bacterial infection (such as septicemia) at any time during labor, or in the 24-hour periods before and after the birth	Respiratory distress starting more than 4 hours after birth
Suspected or confirmed infection in another neonate in case of a multiple pregnancy	Neonatal epileptic seizures
	Need for mechanical ventilation in a term neonate
	Signs of shock
**Non-red flags**	
Invasive group B streptococcal infection in a previous neonate	Altered behavior, -responsiveness or -muscle tone
Maternal group B streptococcal colonization, bacteriuria or infection in the current pregnancy	Feeding difficulties (feed refusal, gastric retention, vomiting, distended abdomen)
Suspected or confirmed rupture of membranes without contractions for more than 24 hours in a term birth	Apnea and bradycardia
Preterm birth following spontaneous labor (before 37 weeks' gestation)	Signs of respiratory distress (tachypnea, moaning, retractions, nasal flaring)
Suspected or confirmed rupture of membranes for more than 18 hours in a preterm birth	Hypoxia (for example, central cyanosis or reduced oxygen saturation level)
Intrapartum fever higher than 38°C or suspected or confirmed chorioamnionitis	Neonatal encephalopathy
	Need for cardio-pulmonary resuscitation
	Need for mechanical ventilation in a preterm neonate
	Persistent pulmonary hypertension
	Temperature abnormality (lower than 36°C or higher than 38°C) unexplained by environmental factors
	Local signs of infection (for example, affecting the skin or eyes)

Intervention EOS calculator: Using the EOS calculator application, between 0-24 hours after birth, maternal EOS risk factors combined with the results of physical examination of the newborn are used to assign a risk category and accompanying clinical recommendation based on estimated EOS incidence (low: <0.65; intermediate: 0.65-1.54; high:> 1.54 per 1000 live newborns) for each newborn at-risk for an infection. The EOS calculator results are used to guide a clinical management decision on performing either a diagnostic work-up and start of empiric intravenous antibiotics for (suspected) EOS, or a conservative approach with routine

controls of vital parameters (heart rate, respiratory rate, and temperature every 3 hours) by the nurse. In case of routine controls, re-evaluation of physical appearance by a pediatric resident or pediatrician will take place within 24 hours postpartum. As an additional safety precaution in our study, the newborns will be observed for 24 hours using vital parameters. In case antibiotics are started, the need for further treatment is assessed after 24-36 hours of treatment depending on blood culture results, infection parameters, and clinical condition of the newborn. Discontinuation of antibiotics and discharge is at the discretion of the treating physician.

4. STUDY POPULATION

4.1 Population (base)

Newborns are eligible for participation if they have a postmenstrual age of 34 weeks or more, are 0-24 hours old, and in case at least one EOS risk factor or clinical sign of infection (suspected of EOS) is present within the first 24 hours of life.

Ten centers will include patients (five randomized to NVK guideline, five to EOS calculator use). Each center is supposed to include around 183 patients. Per center there are between 1500 and 2500 live births a year. The number of eligible patients is estimated to be between 300/year (center with 1500 live births/year and 20% suspected EOS) and 500/year (2500 live births/year and 20% suspected EOS). With an average number of patients of 2000 per center, a baseline suspected EOS of 20%, and at least 50% participation rate of eligible patients, the average number of patients per center that can participate in a one-year period is: 2000 x 0.2 x 0.5 x1= 200 patients. This is more than enough for the needed 183 per center during an 18-month inclusion period. In the Netherlands there are currently no competing trials in this patient population.

4.2 Inclusion criteria

In order to be eligible to participate in this study, a subject must meet all of the following criteria:
- postmenstrual age of 34 weeks or more;
- age between 0-24 hours;
- at least one EOS risk factor or clinical sign of infection (suspected of EOS) present within the first 24 hours of life (Table 1);
- parental/guardian consent.

4.3 Exclusion criteria

A potential subject who meets any of the following criteria will be excluded from participation in this study:
- major congenital anomalies;
- language barrier (lack of effective communication or whenever it hinders understanding).

4.4 Sample size calculation

Superiority: In the control group we expect 40% antibiotic therapy in newborns at risk for EOS. To reduce this to 25% (reduction 15%) by use of the EOS calculator, (80% power, ICC 0,0025, alpha 0,05, 10 hospitals) a total sample size of 330 is required.

Non-inferiority: In the control group and the intervention group, we expect that 10% of newborn infants will fulfill one of the predefined safety criteria. The non-inferiority margin

is set at 15% (absolute difference 5%). The required sample size is 1640 (per cluster 164) (80% power, ICC 0,0025, alpha 0,025, 10 hospitals). Because both hypotheses regarding superiority (reduction in antibiotic therapy) and non-inferiority (adverse clinical outcome) have to be met, the study sample size equals the largest of the two sample sizes.

The potential drop-out or withdrawal is estimated at 10%. We will include 2x 915 newborn infants (total=1830), which means 183 per cluster. The analyses will be performed both according to the intention-to-treat (ITT) principle and according to a per-protocol (PP) analysis. All participants taking part in the study will be analyzed according to the ITT principle. Protocol deviations will be recorded and participants in whom major protocol deviations have occurred will be excluded from the PP analysis. These analyses will be both performed, since attending physicians are able to opt for clinical management not according to the advice of the assigned study protocol. This will be registered as a protocol violation in the eCRF, with accompanying justification (see chapter 3).

5. INVESTIGATIONAL PRODUCT

5.1 Name and description of investigational product

For this study the EOS calculator was developed as a smartphone application (by everywhereIM, Paasheuvelweg 25, Wing D5, 1105 BP Amsterdam-Zuidoost). For additional information, we refer to the attached documents:

- Technical Documentation EOS calculator;
- Productsheet EOS calculator;
- Post-market surveillance plan;
- VWS CE certificering;
- EU declaration of conformity.

The clinical investigation plan equals this study protocol outlined in this document. There is no detailed user manual because the smartphone application is completely self-explanatory. Below is a short description of its procedure:

1. Sign up using work-affiliated email-address, confirm using confirmation mail;
2. Enter input data for a newborn;
3. Press ‘Show results’;

   The app then displays:
   - the *a priori* EOS risk at birth;
   - the EOS risk for each scenario of clinical status of the newborn with accompanying recommendation regarding medical management;
4. Press ‘Back’ if you want to check or change your inserted data, or
5. Press ‘Save and send’: all inserted data and the recommendation will be sent to your linked email address. During the study, these data will also be sent to the email address of the research nurse. These data will contain a unique identifier for that particular calculation session (not traceable to the newborn), for quality control and monitoring purposes.

These emails are study documents and will be saved for the duration of 15 years after the end of the research period, as is common for all research documents. They will be stored at Tergooi Hospital, the sponsor. All inputted data will be coded and stored at the server of everywereIM as well. This is not traceably information and will not be accessible for third parties. EverywhereIM is ISO- and NEN-certified.

The CE marking and classification of the EOS Calculator app are valid for five years, and was granted in accordance with the Medical Device Directive 93/42/EEG and the system of

GHTF/SG1/N15: 2006 Principles of Medical Devices Classification respectively (see ‘VWS CE certificering’ and ‘Technical Documentation EOS Calculator’ part 2.3). The validity of these documents has been verified at the secretariat of the Medical Research Ethics Committee of the Amsterdam UMC, location Academic Medical Center (on July 20, 2021). All other documents provided are in accordance with article 82 of the Medical Device Regulation.

5.2 Summary of findings from non-clinical studies

The development and validation of two linked predictive models for EOS was described in two clinical papers (14,15), and recently summarized (29). Briefly, these models employ a Bayesian approach. The first model established newborn’s probability of EOS entirely based on the maternal risk factor, or EOS risk at birth (15). EOS risk at birth is calculated using gestational age and maternal variables that are available at the moment of birth. The relationship of each variable to the outcome was analyzed separately and then combined into a multivariate model. The second predictive model for EOS that was developed quantifies how the baseline risk is modified by the newborns clinical examination (14). Three clinical-presentation categories (clinical illness, equivocal, and well appearing) were defined and calculated likelihood ratios for each. Newborns will be stratified based on both their clinical presentation and the three levels of sepsis at birth. And management choices are suggested (no treatment-observation-treat empirically with antibiotics). This all was developed into a Web-based EOS calculator that provides physicians with an explicit risk estimate. The first release was in December 2012.

The process of the web-based EOS calculator has been copied into the app. Validation has been performed to ensure that the app functions properly. More detailed information about technical specifications and the validation of the smartphone application is included in the above mentioned documents (see ‘Technical Documentation EOS Calculator’ chapter 2 and 7).

5.3 Summary of findings from clinical studies

The EOS calculator has been developed and validated on data of over >600,000 newborns born at ≥34 weeks’ gestation. The implementation study then included 204,485 newborns and showed a decrease in antibiotic use from 5.0% to 2.6% (16). EOS incidence and readmissions for EOS did not change after implementation. The EOS calculator has now been evaluated in more than 20 studies, of which 8 with actual implementation. Meta-analysis shows up to an average of 44% less antibiotic use, without indications of negative

consequences (17). Implementation in Tergooi hospital gave a similar result and a Dutch multicenter study shows a similar potential (18). There are no known implementation studies in which the use of the EOS calculator was not effective or proved unsafe. Since the content of the smartphone application is an exact copy of the web-based version, the content has already been validated in America. This study will validate the content for the Dutch population.

5.4 Summary of known and potential risks and benefits

Benefit: Meta-analysis shows up to an average of 44% less antibiotic use, without indications of negative consequences (17).

Risk: We like to address that the use of the EOS calculator is not able to capture all newborns with a proven EOS, comparable to any other guideline. It is important to realize that missed cases does not mean that the missed cases have died or have a worse outcome. It means that antibiotics were not started immediately after birth. The EOS calculator-based strategy is therefore not automatically inadequate or less safe, but it indicates that the calculator relies to a greater extent on clinical observation for 24 hours that is advised by the calculator. Achten et al. reviewed all cases of EOS in a meta-analysis. Among before-after implementation studies, they found 5 of 18 (28%) “missed” cases in cohorts with EOS calculator-based management, compared to 8 of 28 “missed” cases (29%) in cohorts with conventional management strategies. All newborns with “missed” EOS were eventually started on antibiotics. To have no missed cases, we must treat all babies after delivery, which is undesirable because we know that antibiotics early in life do harm.

6. METHODS

6.1 Study parameters/endpoints

6.1.1 Main study parameter/endpoint

Co-primary superiority outcome: The proportion of patients that started antibiotic therapy for suspected and, or proven EOS in the first 24 hours after birth.

Co-primary non-inferiority outcome: A composite non-inferiority outcome regarding the presence of one or more of the following four predefined safety criteria, namely 1) the need for any respiratory support, and/or 2) the need for an intravascular fluid bolus for hemodynamic instability due to sepsis, and/or 3) referral to a Neonatal Intensive Care Unit for sepsis treatment, and/or 4) proven EOS.

- Respiratory support is defined as any form of respiratory support (invasive ventilation, continuous positive airway pressure (CPAP), high flow nasal cannula (HFNC), low flow oxygen) during the first week of life.
- Intravascular fluid bolus is defined as the intravenous administration of a fluid bolus of 10ml/kg within 15-30 minutes. It is the first step in the treatment of hemodynamically unstable newborns due to sepsis.
- Proven EOS is defined as a blood or cerebrospinal fluid (CSF) culture obtained within 72 hours after birth growing a pathogenic bacterial species.

The mentioned co-primary outcome criteria overlap with non EOS causes. For example, prematurity is also associated with the need for respiratory support or asphyxia with the need for intravascular fluid administration. It is for this that a randomization trial was chosen. We do not expect that a reduction in antibiotic exposure by using the EOS calculator will lead to significantly more of these outcomes. Because the trial is not blinded, there is a change that (unconscious) bias may occur. We expect that this will be limited by the cluster-randomized design on the one hand, and by the relatively objective criteria, on the other hand.

Furthermore, we decided for a composite non-inferiority outcome, based on four predefined criteria, since the incidence of each individual criterion is very low.

6.1.2 Secondary study parameters/endpoints

- The total duration of antibiotic therapy.
- The proportion of antibiotic therapy started for suspected and, or proven EOS if symptoms started between 24-72 hours after birth.

- Quality of life: To get an impression of the impact of (suspected) early-onset sepsis on the quality of life (QoL) of both parents/guardians and their child, parents/guardians will be asked to fill in a questionnaire on day 14 after birth. Our questionnaire is based on (parts of) two questionnaires, which were used in different studies assessing the effect of early intravenous-to-oral antibiotic switch (30), and the influence of resiliency and stress on parental/guardian perspective of the future QoL of their newborns (31). The first part collects information on medical factors after hospitalization (side effects, number of medical visits, readmission, and medication use) and basic needs of the newborn (sleep quality of the patient, (breast)feeding success rate). The second part addresses the subjective parental/guardian evaluation of the impact of newborns' admission, and parental/guardian projection of future QoL of their newborn. The last part of the questionnaire collects demographic data. We hypothesize that the maternity period is experienced more positive if mother and child go home quickly, because of less hinder to take care of their child (for example, earlier initiation of breastfeeding), and less stress (for example, emotionally or financially). The questionnaire is designed in close collaboration with Care4Neo. Care4Neo (formerly Association of Parents of Incubator Children) represents the interests of parents/guardians whose child is admitted to a hospital immediately after birth.

6.2 Randomization, blinding and treatment allocation

Randomization will take place at cluster level for either the EOS calculator or control (antibiotics administration according to NVK directive usual care). Centers will be randomized as clusters using a computer-generated algorithm. After randomization, the hospital will also switch to the protocol assigned after randomization (NVK guideline or EOS calculator use). This assigned protocol is standard care for newborns with suspected EOS during the study period in that hospital. This is independent of participating in this study (see chapter 3). Thus, hospitals nor physicians are blinded for the assigned study protocol. However, physicians may always opt for clinical management not according to the assigned study protocol.

6.3 Study procedures

For all included newborns, maternal and neonatal data on risk factors will be collected. The newborn's date and time of birth will be collected because the most important collected parameters are time dependent and need these date and time of birth as a reference. Physical examinations of the newborn will be performed at least twice (at birth and before discharge), and more often if necessary (depending on clinical findings). Finally, for

participating newborns, a single (online) questionnaire will be sent (via Castor EDC) to the parents/guardians to fulfill on day 14 after birth. The questionnaire includes a minimum of 22 questions, which may expand up to 32 questions (since some questions are dependent of other questions). It will take about 10-15 minutes to complete.

For newborns without antibiotic therapy, clinical observation for at least 12-24 hours will be performed: vital parameters (heart rate, respiratory rate, and temperature) will be measured 1, 3, 6, 9, 12, 15, 18, 21 and 24 hours after birth. This is part of standard medical treatment. Data on physical examinations will be collected. Data on readmission for suspected or proven EOS will be collected.

For newborns with intravenous antibiotic therapy, two blood samples will be taken: during insertion of the intravenous cannula before starting antibiotics (blood culture; blood cell count, CRP), and approximately 24-36 hours after the start of antibiotics by capillary or vena puncture (CRP). This is part of standard medical treatment. Duration of hospital stay will be at least 24-36 hours, could be extended depending on the clinical condition and blood culture results and non-EOS factors. During admission the need for any kind of respiratory support and the need for intravascular fluid administration will be recorded daily. Referral to a neonatal intensive care unit (NICU) will be recorded and relevant clinical data during NICU admission will be obtained by the main investigator or the research nurse/assistant of the participating hospital. Data on sort and duration of antibiotic therapy and blood culture results will be collected.

Figure 1. Timeline - Recruitment to Completed eCRF

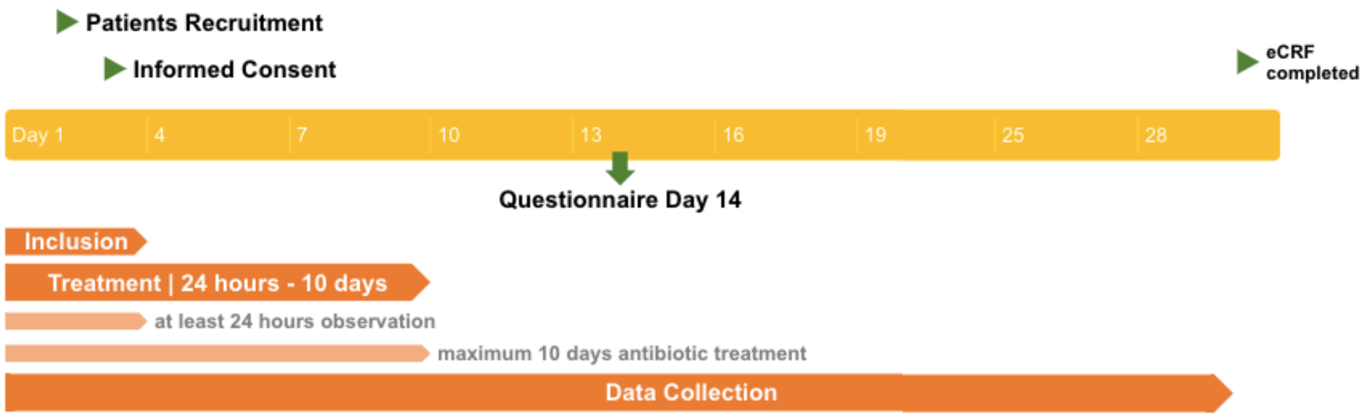

6.4 Withdrawal of individual subjects

Subjects can leave the study at any time for any reason if they wish to do so without any consequences. The investigator can decide to withdraw a subject from the study for urgent medical reasons.

6.5 Replacement of individual subjects after withdrawal

Subjects will not be replaced after withdrawal because we already taken into account in the sample size calculations a 10% drop-out.

6.6 Follow-up of subjects withdrawn from treatment

N/A

6.6.1 Premature termination of the study

Interim analyses for safety are planned after outcome data for the first 200 participants in either arm (in total 400) have come available. This will be repeated at a total of 900 participants. Based on the results of the safety interim analysis the DSMB may advise to stop the trial. The sponsor will inform the accredited METC in case of termination and the reason for it within 15 days.

7. SAFETY REPORTING

7.1 Temporary halt for reasons of subject safety

In accordance with section 10, subsection 4, of the WMO, the sponsor will suspend the study if there is sufficient ground that the continuation of the study will jeopardize the subject health or safety. The sponsor will notify the accredited METC without undue delay of a temporary halt including the reason for such an action. The study will be suspended pending a further positive decision by the accredited METC. The investigator will take care that all subjects are kept informed.

7.2 AEs and SAEs

All (serious) adverse events reported spontaneously by the parent(s)/guardian(s) of the subject or observed by the investigator, or his staff will be recorded up until and including day 14 after birth. This period is consistent with the follow-up time and reasonably includes complications that may be linked to initial EOS management, such as a different course of EOS or readmission. In the unusual case that a newborn still receives antibiotic therapy after day 14 of life because of EOS (or a SAE), (serious) adverse events will be reported until the end of therapy.

7.2.1 Adverse events (AEs)

Adverse events are defined as any undesirable experience, symptom, or medical condition occurring to a subject during the study, whether or not considered related to the trial procedure.

Not considered AEs are:

- regurgitation;
- colic;
- abnormal laboratory values, not related to neonatal early-onset infection, unless they are considered clinically significant or require therapy (except phototherapy for hyperbilirubinemia, and ferrous fumarate or erythrocyte transfusions for anemia);
- abnormal test results, other than blood cultures, unless they are considered clinically significant or require therapy.

7.2.2 Serious adverse events (SAEs)

A serious adverse event is any untoward medical occurrence or effect that

- results in death;
- is life threatening (at the time of the event);
  - except, if it concerns neonatal (advanced) life support directly after birth;
  - except if it concerns surfactant therapy;
  - except referral to a NICU:
    - (need for mechanical ventilation because of) surfactant therapy;
    - insertion of central venous lines;
    - respiratory support for prematurity.
- requires hospitalization or prolongation of existing inpatients' hospitalization;
  - unless a newborn will start with antibiotic therapy for (suspected) EOS during the observation period or after discharge, but within 72 hours after birth. See also 5.4.;
  - unless it is due to prematurity.
- results in persistent or significant disability or incapacity; or
- any other important medical event that did not result in any of the outcomes listed above due to medical or surgical intervention but could have been based upon appropriate judgement by the investigator.

Referral to a NICU will be included in the electronic Case Report Form (eCRF), since this is one of the non-inferiority criteria.

Each study site will report all SAEs to the coordinating investigators within 24 hours after becoming aware of the event. The coordinating investigators will report all SAEs to the sponsor without undue delay after obtaining knowledge of the events. The sponsor will report the SAEs through the web portal *ToetsingOnline* to the accredited METC that approved the protocol, within 7 days of first knowledge for SAEs that result in death or are life threatening followed by a period of a maximum of 8 days to complete the initial preliminary report. All other SAEs will be reported within 15 days after the sponsor has first knowledge of the serious adverse events.

7.3 Annual safety report

The sponsor will submit, once a year throughout the clinical trial, a safety report to the accredited METC.

This safety report consists of:

- a list of all suspected (unexpected or expected) serious adverse events, along with an aggregated summary table of all reported serious adverse events, ordered by organ system, per study;
- a report concerning the safety of the subjects, consisting of a complete safety analysis.

7.4 Follow-up of (serious) adverse events

All (S)AEs will be followed up until they have abated, or until a stable situation has been reached. Depending on the event, follow-up may require additional tests or medical procedures as indicated, and/or referral to the general physician or a medical specialist. SAEs need to be reported till end of study within the Netherlands, as defined in the protocol (see 7.2).

7.5 Data Safety Monitoring Board (DSMB) / Safety Committee

An independent DSMB will monitor this trial and consists of several members with expertise in the relevant fields of paediatrics, epidemiology and statistics. The composition of the DSMB is outlined in the DSMB Charter. This document also includes statements regarding conflict of interest by the members of the board and an outline of tasks and responsibilities.

The safety of trial participants is of primary concern for the DSMB. The DSMB will meet to discuss the safety review after the outcome data of the first 400 patients (200 in the control arms and 200 in the intervention arm) and 900 patients have become available. Additional reviews will be conducted at the DSMBs request. The DSMB can advise to terminate the study prematurely in case an interim analysis shows clear harm of either one of the interventions or due to external evidence. A formal interim analysis for efficacy (inferential testing) will not be conducted. The DSMB will not be blinded when first assessing the data.

Criteria on which the DSMB may decide to terminate the trial are described in the DSMB charter (see ‘K5. DSMB Charter’). The advice(s) of the DSMB will be sent to the principal investigator and the trial methodologist. Should the principal investigator decide not to fully implement the advice of the DSMB, the principal investigator will send the advice to the reviewing METC, including a note to substantiate why (part of) the advice of the DSMB will not be followed.

8. STATISTICAL ANALYSIS

8.1 Primary study parameters

The analyses will be performed according to the Intention-To-Treat and Per Protocol principle (as secondary analysis). The primary analysis will estimate the relative risk with 95% confidence intervals and p-values for the primary outcomes, using a generalized linear mixed model with log link and binomial distribution, with random intercepts and random slopes per cluster as appropriate. A significance level of 5% will be used.

Both superiority for the proportion of patients that started antibiotic therapy and non-inferiority for adverse clinical outcome must be shown to conclude the effectiveness of the EOS calculator. The use of EOS calculator will be considered superior when it leads to at least a 15% absolute reduction in the proportion of patients who started antibiotic therapy. We expect 10% neonatal complications in the control group. The use of the EOS calculator will be considered non-inferior when the upper bounds of a 95% confidence interval of the relative risk of complications does not exceed 1.5 (comparable to a 15% complication rate).

8.2 Secondary study parameters

Dichotomous secondary outcomes will be analysed using the same method as the primary outcome. Continuous data of secondary outcomes will be assessed using a linear mixed model to estimate mean differences, with random intercepts and slopes as appropriate. Median differences will be calculated as appropriate.

8.2.1 Quality of life

To get an impression of the impact of (suspected) early-onset sepsis on the quality of life (QoL) of both parents/guardians and their child, parents/guardians will be asked to fill in a questionnaire on day 14. See also 6.1.2. Outcome reporting will be mainly descriptive. Mixed models can be used to further explore the effect of treatment and center if a trend is observed for some of the questions. Mean scores and standard deviations are calculated for composite indicators. We use t-tests to evaluate differences in mean scores between groups for normally distributed data. The significance level is set at an alpha value of <.05.

8.3 Interim analysis

There are two interim analyses planned, which are based on safety. However, efficacy may be associated with safety. Interim safety review will use descriptive statistics only. See ‘K5. DSMB Charter’ for more details.

9. ETHICAL CONSIDERATIONS

9.1 Regulation statement

The study will only start after written approval from the Independent Ethics Committee (IEC), which operates according to ICH GCP guidelines. The investigator is responsible for submission to and communication with the IEC.
The study will be conducted in accordance with the principles of the declaration of Helsinki (October 2008), the ICH GCP guidelines (CPMP/ICH/135/95), the Regulations on Medical Research involving Human subjects (Medical Research involving Human subjects Act, Wet Medisch Wetenschappelijk Onderzoek met mensen WHO, 1999), and the Medical Device Regulation (MDR/2017/745). The investigator will not implement any amendment of the protocol without IEC approval, except when changes involve only logistical or administrative aspects of the study.

9.2 Recruitment and consent

The study will be conducted according to the “Code of conduct relating to expressions of objection by minors participating in medical research” approved by the Board of the Dutch Association of Paediatric Medicine (NVK) on 21 May 2001, see Appendix I. Parents or guardians are free to decide to withdraw from the study at any stage, and for any reason, without prejudicing their child’s further treatment.

Recruitment of patients

Within the first 24 hours after birth parents or guardians of eligible newborns (whom are at risk for EOS) will be approached by local principal investigators or research nurses, which are well trained (according to ICH GCP) and highly engaged in the study. The parent(s)/guardian(s) will receive more extensive information (in Dutch or English) in the patient information and consent form. Prior to entering the study, the investigator will explain to the parent(s)/guardian(s) the nature of the study, its purpose, procedures, expected duration, and the benefits and risks involved in study participation.

The patient will be given sufficient time to read the patient information and the informed consent form and has the opportunity to ask questions before signing the informed consent form. The investigator or research nurse will answer all study-related questions. The right to withdraw from the study at any time without prejudice will be clearly communicated. After this explanation and before any study-specific procedures have been performed, the patient, as well as the investigator/research nurse, will voluntarily sign and date an informed consent form.

The policy (observation and/or antibiotic therapy) is not conducted by participating in the study, since the assigned protocol is considered standard care for newborns with suspected EOS during the study period. (See also chapter 3 and 6.2.) No additional painful procedures will be performed. These things will make it more likely parents/guardians will give permission to participate.

A newborn is at risk for EOS if at least one maternal risk factor or neonatal clinical sign is present (in accordance with the NVK guideline, listed in table 1). After determining eligibility, the advice of either the NVK guideline or the EOS calculator is followed (depending on randomization). The observation period will be at least 12 hours for the control group (NVK guideline) and 24 hours for the intervention group (EOS calculator), which consists of routine controls of vital parameters (heart rate, respiratory rate, and temperature) every 3 hours by the nurse, and a minimum of two physical examinations (within 4 hours after birth and before discharge) by the physician.

Conduct consent

We use the deferred consent concept for participation. In this cluster randomized trial, the compared groups both consist of standard care (i.e. NVK guideline or EOS calculator, see chapter 3 and 6.2). Consent for standard care is not required. Consent for data collection of standard care and completing a questionnaire will be gained by the investigator/research nurse. Most of the time this will be within the first 24 hours after birth. We may gain consent in a later phase (more than 24 hours after birth), since we think it is reasonable and achievable to gain consent during normal working hours. No data will be collected from patients who refuse to participate.

There are two consent forms: one for permission to use data of the child, which has to be signed by both parents or guardians, and one for permission to use data of the mother, which has to be signed by the mother only. The consent forms consist of two pages. The first page contains general statements and specific statements about participating in the

study. On this page parents or guardians will be asked to give consent to collect data from the electronic medical records of mother and child (see chapter 3 and the patient information form), and to fill in one questionnaire about quality of life on day 14. Signing the form by (both) parent(s)/guardian(s) automatically means that they agree with these statements. There are three optional statements included on the first page, where they can choose whether they give permission. Parent(s)/Guardian(s) will be asked whether they give permission to save personal data from mother and child for a longer period to use these for future research, and to possible study-related follow-up (questionnaires) in the future (after day 14).

9.3 Objection by minors or incapacitated subjects

The study will be conducted according to the “Code of conduct relating to expressions of objection by minors participating in medical research” approved by the Board of the Dutch Association of Paediatric Medicine (NVK) on 21 May 2001, see Appendix I. Parents or guardians are free to decide to withdraw from the study at any stage, and for any reason, without prejudicing their child’s further treatment.

9.4 Benefits and risks assessment, group relatedness

This study will have negligible risks and minimal burden.

Burden: -Newborns with antibiotic therapy: A intravenous cannula will be inserted for antibiotic treatment. According to current Dutch guidelines two blood samples will be taken: directly after birth during insertion of the intravenous cannula (blood culture; at least 1 mL, blood cell count, CRP, procalcitonin), and 24 hours after birth by vena puncture (CRP, procalcitonin). This is part of standard medical treatment. Hospital stay will be at least 48 hours, could be extended depending on the clinical condition and blood culture results (ranging from 36 hours to 10 days). - Newborns without antibiotic therapy: No blood samples taken. Clinical observation for at least 12-24 hours. This is part of standard medical treatment. - All newborns: Physical examinations will be performed at least twice (at birth and before discharge), and more often if necessary (depending on clinical findings). One questionnaire will be sent to the parents/guardians to fill in on day 14, this is extra for this study. The questionnaire includes a minimum of 22 questions, which may expand up to 32 questions (since some questions are dependent of other questions). It will take about 10-15 minutes to complete.

Risks: The risks associated with participation are very low because neonates will be closely observed when no antibiotics will be prescribed.

Benefits: More specific risk analysis. Not all subjects will benefit personally from study participation. When no antibiotics will be prescribed: no separation of mother and child, no painful procedures, and a shorter hospital stay (including fewer costs).
Group relatedness: This study can only be performed with this specific group of patients, as it will provide age-specific and risk factor-specific data that cannot be obtained otherwise.

9.5 Compensation for injury

This study is exempt from the WMO insurance obligation as there are no significant risks attributable to participation in this study. This is in accordance with article 7, paragraph 6 of the WMO.

10. ADMINISTRATIVE ASPECTS, MONITORING AND PUBLICATION

10.1 Handling and storage of data and documents

Data will be handled and analysed confidentially. Data will be collected in an electronic Case Report Form (eCRF) in Castor EDC to document eligibility, safety and efficacy parameters, and parameters necessary to evaluate endpoints. Each CRF will be completed on-site by the investigator or an authorized staff member. The data is handled confidentially, and a unique patient study number will be used to link the data to the subject in compliance with *EU General Data Protection Regulation and the Dutch Act on Implementation of the General Data Protection Regulation (in Dutch: Uitvoeringswet AVG, UAVG)*. All eCRF entries must be based on source documents.

Data of the EOS calculator will be sent to the work-affiliated email address of the authorized persons who use the calculator. A copy of this email will automatically be sent to the main research nurse. Every email contains a unique token. The email contains instructions for copying the results and the token to the electronic patient files. The results of the EOS calculator are included in the eCRF; there is no direct link from the smartphone application to Castor EDC.

Data of the questionnaire will be obtained online. This will be directly linked to Castor EDC. For detailed information about the questionnaire see 6.1.2, 6.3, 8.2.1 and 9.2.

Individual subject medical information obtained as a result of this study is considered confidential and disclosure to third parties is prohibited. Such medical information may be given to the subject's physician or to other appropriate medical personnel responsible for the subject's wellbeing. Data generated as a result of this study are available for inspection on request by the participating physicians, the IEC and the regulatory health authorities, including external site audits and inspections.

All study-related documents will be stored online at https://www.zorgevaluatienederland.nl/eos. Investigators of the participating hospitals will get access to this environment and all documents. General information about the study is available for everyone at the above-mentioned website, without the need of a password.

Every participating hospital has an Investigator Site File, located at their own protected server. The investigators will find the newest main documents of the study at https://www.zorgevaluatienederland.nl/eos. It is the responsibility of the coordinating

investigators to keep these documents up to date. The investigators at the participating hospitals are responsible for keeping local agreements and related documents up to date. The Trial Master File (TMF) is located at the protected server of the sponsor, Tergooi hospital. The TMF falls under the responsibility of the principal investigator, prof. dr. F.B. Plötz.

Documents and data will be stored for 15 years. Data management will done be according to the FAIR data principles (FORCE11). Our data will be findable, accessible, interoperable and reusable. Results will be shared as scientific publications, where possible via open access channels. Where possible, data will be shared in accordance with guidelines to promote further improvements and optimisation.

10.2 Monitoring and Quality Assurance

We qualified the risk of the study as negligible risk, based on the guideline by the NFU (Dutch Federation of University Medical Centers) about quality insurance in human research. Although this study will use a smartphone application, which is considered a medical device, the content derived from web-based software and is widely used and validated in over 600,000 newborns without any complication (16). See chapter 5 and 11 for details. Steps to be taken to ensure the accuracy and reliability of data include the selection of qualified investigators and appropriate study centers, review of protocol procedures with the investigator before the study, and yearly site monitoring visits by the trial agency in all participating centers during the study. Data collected on the eCRF will be verified for accuracy. If necessary, queries will be sent to the investigational site on a regular base to clarify the data in eCRF. The investigator should answer data queries within the specified timeline. Details are included in the monitoring plan, which will be managed by the trial agency. This document is attached.

10.3 Amendments

A 'substantial amendment' is defined as an amendment to the terms of the METC application, or to the protocol or any other supporting documentation, that is likely to affect to a significant degree:

- the safety or physical or mental integrity of the subjects of the trial;
- the scientific value of the trial;
- the conduct or management of the trial; or
- the quality or safety of any intervention used in the trial.

All substantial amendments will be notified to the METC and to the competent authority. Non-substantial amendments will not be notified to the accredited METC and the competent authority but will be recorded and filed by the sponsor.

10.4 Annual progress report

The investigator will submit a summary of the progress of the trial to the accredited METC once a year. Information will be provided on the date of inclusion of the first subject, numbers of subjects included and numbers of subjects that have completed the trial, serious adverse events, other problems, and amendments.

10.5 Temporary halt and (prematurely) end of study report

The sponsor will notify the accredited METC and the competent authority of the end of the study within a period of 90 days. The end of the main study is defined as the last patient's last questionnaire is completed and the record is closed (at a maximum of 28 days after birth).

The sponsor will notify the METC immediately of a temporary halt of the study, including the reason for such an action. In case the study is ended prematurely, the sponsor will notify the accredited METC and the competent authority within 15 days, including the reasons for the premature termination. Within one year after the end of the study, the investigator/sponsor will submit a final study report with the results of the study, including any publications/abstracts of the study, to the accredited METC and the competent authority.

10.6 Public disclosure and publication policy

The study will be considered for publication or presentation at (scientific) symposia or congresses. Authorship will follow the guidelines defined by the International Committee of Medical Journal Editors (http://www.icmje.org). Since patient data are recorded anonymously, patient privacy will be guaranteed. The results will be used to improve and publish new guidelines.

11. STRUCTURED RISK ANALYSIS

11.1 Potential issues of concern

a. Level of knowledge about mechanism of action

N/A

b. Previous exposure of human beings with the test product(s) and/or products with a similar biological mechanism

See chapter 5.

c. Can the primary or secondary mechanism be induced in animals and/or in *ex-vivo* human cell material?

N/A

d. Selectivity of the mechanism to target tissue in animals and/or human beings

N/A

e. Analysis of potential effect

See chapter 5.

f. Pharmacokinetic considerations

N/A

g. Study population

See chapter 4.

h. Interaction with other products

N/A

i. Predictability of effect

See chapter 5.

j. Can effects be managed?

N/A

11.2 Synthesis

The change of study-related injury is minimal. Many experts, including a methodologist, a quality advisor, and several paediatricians with a great amount of research experience, were involved during development of the protocol. The research design is not complex and does not contain harmful or painful procedures. Seven (out of ten) hospitals are already known with this kind of research because of our earlier multicenter study(13,20). All researchers in our research group are experienced.

For this study the EOS calculator was developed as a mobile application (by everywhereIM), including CE marking. It is concerned a medical device for health care professionals. Chapter 11.1 was skipped, because all applicable information about this medical device is given in chapter 5.

Though this application is just registered and is not yet used in practice, its web-based software version is widely used and validated in over 600,000 newborns already(16). It will be used within the same indication, and we will not use it in combination with another product.

There is always a chance of protocol deviation or violation. In earlier research, deviation from the current Dutch guidelines has been seen(13), while adherence to the EOS calculator was high(18). However, local investigators are encouraged to follow the assigned protocol. If attending physicians opt for clinical management not according to the assigned study protocol, this will be recorded with deviations being noted with accompanying justification.

Newborns are a vulnerable research population. To ensure safety of trial participants we established a DSMB. Care4Neo and trial agency ‘Zorgevaluatie Nederland’ are also involved.

We qualified the overall risk of the study as “negligible risk”, based on the guideline by the NFU (Dutch Federation of University Medical Centers) about quality insurance in human research (32).

12. REFERENCES

1. Shane AL, Sanchez PJ, Stoll BJ. Neonatal sepsis. *Lancet* 2017; 390:1770–80.
2. Mukhopadhyay, S.; Puopolo KM. Neonatal Early-Onset Sepsis : Epidemiology and Risk. 2015; 16.
3. Simonsen KA, Anderson-Berry AL, Delair SF, Dele Davies H, Davies HD. Early-onset neonatal sepsis. *Clin Microbiol Rev* 2014; 27:21–47.
4. Klingenberg C, Kornelisse RF, Buonocore G, Maier RF, Stocker M. Culture-Negative Early-Onset Neonatal Sepsis — At the Crossroad Between Efficient Sepsis Care and Antimicrobial Stewardship. *Front Pediatr* 2018; 6:1–9.
5. Cantey JB, Wozniak PS, Pruszynski JE, Sanchez PJ. Reducing unnecessary antibiotic use in the neonatal intensive care unit (SCOUT): a prospective interrupted time-series study. *Lancet Infect Dis* 2016; 16:1178–84.
6. van Herk W, Stocker M, van Rossum AMC. Recognising early onset neonatal sepsis: an essential step in appropriate antimicrobial use. *J Infect* 2016; 72:S77–82.
7. Korpela K, Zijlmans MAC, Kuitunen M, Kukkonen K, Savilahti E, Salonen A, et al. Childhood BMI in relation to microbiota in infancy and lifetime antibiotic use. *Microbiome* 2017; 5:1–9.
8. Low JSY, Soh S, Lee YK, Kwek KYC, Holbrook JD, Beek EM Van Der. Europe PMC Funders Group Ratio of Klebsiella / Bifidobacterium in Early Life Correlates with Later Development of Paediatric Allergy. *Benef Microbes* 2017; 8:681–95.
9. Sidney E. Zven, Apryl Susi, Edward Mitre CMN. Association Between Use of Multiple Classes of Antibiotic in Infancy and Allergic Disease in Childhood. *JAMA Pediatr* 2019.
10. Cotten CM. Adverse Consequences of Neonatal Antibiotic Exposure. *Physiol Behav* 2016; 28:141–9.
11. NVOG (Nederlandse Vereniging voor Obstetrie en Gynaecologie), NVK (Nederlandse Vereniging Kindergeneeskunde). Preventie en behandeling van early-onset neonatale infecties (Adaptatie van de NICE-richtlijn). 2017 p. 1–94.
12. National Institute for Health and Clinical Excellence. Neonatal infection (early onset): Antibiotics for prevention and treatment [Internet]. Clinical Guideline. 2012 p. 1–40. Available from: https://www.nice.org.uk/guidance/cg149/resources/neonatal-infection-early-onset-antibiotics-for-prevention-and-treatment-35109579233221
13. van der Weijden BM, Achten NB, Bekhof J, Evers EE, Berk M, Kamps AWA, et al. Multicentre study found that adherence to national antibiotic recommendations for neonatal early-onset sepsis was low. *Acta Paediatr Int J Paediatr* 2020:1–8.
14. Escobar GJ, Puopolo KM, Wi S, Turk BJ, Kuzniewicz MW, Walsh EM, et al. Stratification of risk of early-onset sepsis in newborns ≥ 34 weeks' gestation.

Pediatrics 2014; 133:30–6.

15. Puopolo KKM, Draper D, Wi S, Newman TB, Zupancic J, Lieberman E, et al. Estimating the probability of neonatal early-onset infection on the basis of maternal risk factors. *Pediatrics* 2011; 128:e1155-63.
16. Kuzniewicz MW, Puopolo KM, Fischer A, Walsh EM, Li S, Newman TB, et al. A quantitative, risk-based approach to the management of neonatal early-onset sepsis. *JAMA Pediatr* 2017; 171:365–71.
17. Achten NB, Klingenberg C, Benitz WE, Stocker M, Schlapbach LJ, Giannoni E, et al. Association of Use of the Neonatal Early-Onset Sepsis Calculator with Reduction in Antibiotic Therapy and Safety: A Systematic Review and Meta-analysis. *JAMA Pediatr* 2019; 173:1032–40.
18. Achten NB, Dorigo-Zetsma JW, van der Linden PD, van Brakel M, Plötz FB. Sepsis calculator implementation reduces empiric antibiotics for suspected early-onset sepsis. *Eur J Pediatr* 2018; 177:741–6.
19. NICE. Neonatal infection: antibiotics for prevention and treatment NICE guideline. 2021:NICE Guideline No 195.
20. van der Weijden BM, Achten NB, Bekhof J, Evers EE, van Dongen O, Rijpert M, et al. Neonatal early-onset sepsis calculator recommended significantly less empiric antibiotic treatment than national guidelines. *Acta Paediatr Int J Paediatr* 2020; 109:2549–51.
21. Achten, Niek B.; Klingenberg, C.; Plötz FB. Neonatal Early-Onset Sepsis Calculator and Antibiotic Therapy-Reply. *JAMA Pediatr* 2020; 174:508–9.
22. Kerste M, Corver J, Sonnevelt MC, van Brakel M, van der Linden PD, M. Braams-Lisman BA, et al. Application of sepsis calculator in newborns with suspected infection. *J Matern Neonatal Med* 2016; 29:3860–5.
23. Achten N, Zonneveld R, Tromp E, Plötz F. Association between sepsis calculator and infection parameters for newborns with suspected early onset sepsis. *J Clin Neonatol* 2017; 6:159.
24. Achten NB, Visser DH, Tromp E, Groot W, van Goudoever JB, Plötz FB. Early onset sepsis calculator implementation is associated with reduced healthcare utilization and financial costs in late preterm and term newborns. *Eur J Pediatr* 2020; 179:727–34.
25. Achten NB, Dorigo-Zetsma JW, van Rossum AMC, Oostenbrink R, Plötz FB. Risk-based maternal group B Streptococcus screening strategy is compatible with the implementation of neonatal early-onset sepsis calculator. *Korean J Pediatr* 2020; 63:406–10.
26. Benitz WE, Achten NB. Finding a role for the neonatal early-onset sepsis risk calculator. *EClinicalMedicine* 2020; 19:100255.

27. Benitz WE, Achten NB. Technical assessment of the neonatal early-onset sepsis risk calculator. *Lancet Infect Dis* 2021; 21:e134–40.
28. Achten NB, Plötz FB, Klingenberg C, Stocker M, Bokelaar R, Bijlsma M, et al. Stratification of Culture-Proven Early-Onset Sepsis Cases by the Neonatal Early-Onset Sepsis Calculator: An Individual Patient Data Meta-Analysis. *J Pediatr* 2021.
29. Kuzniewicz MW, Walsh EM, Li S, Fischer A, Escobar GJ. Development and implementation of an early-onset sepsis calculator to guide antibiotic management in late preterm and term neonates. *Jt Comm J Qual Patient Saf* 2016; 42:232–9.
30. Keij FM, Kornelisse RF, Hartwig NG, Mauff K, Poley MJ, Allegaert K, et al. RAIN study: A protocol for a randomised controlled trial evaluating efficacy, safety and cost-effectiveness of intravenous-to-oral antibiotic switch therapy in neonates with a probable bacterial infection. *BMJ Open* 2019; 9:1–8.
31. Ferrand A, Gorgos A, Ali N, Payot A. Resilience Rather than Medical Factors: How Parents Predict Quality of Life of Their Sick Newborn. *J Pediatr* 2018; 200:64-70.e5.
32. Van NF, Centra UM. Richtlijn Kwaliteitsborging Mensgebonden Onderzoek 2019. 2019.
